# Transcriptome‐wide analysis of circRNA and RBP profiles and their molecular relevance for GBM

**DOI:** 10.1002/1878-0261.70005

**Published:** 2025-02-26

**Authors:** Julia Latowska‐Łysiak, Żaneta Zarębska, Marcin P. Sajek, Adriana Grabowska, Alessia Buratin, Paweł Głodowicz, Julia O. Misiorek, Konrad Kuczyński, Stefania Bortoluzzi, Marek Żywicki, Jan G. Kosiński, Agnieszka Rybak‐Wolf, Rafał Piestrzeniewicz, Anna M. Barciszewska, Katarzyna Rolle

**Affiliations:** ^1^ Department of Molecular Neurooncology Institute of Bioorganic Chemistry, Polish Academy of Sciences Poznań Poland; ^2^ RNA Bioscience Initiative, University of Colorado School of Medicine Aurora CO USA; ^3^ Department of Biochemistry and Molecular Genetics University of Colorado School of Medicine Aurora CO USA; ^4^ Institute of Human Genetics, Polish Academy of Sciences Poznań Poland; ^5^ Department of Molecular Medicine University of Padua Italy; ^6^ Faculty of Chemistry Adam Mickiewicz University Poznań Poland; ^7^ Center for Advanced Technology Adam Mickiewicz University Poznań Poland; ^8^ Department of Computational Biology Institute of Molecular Biology and Biotechnology, Adam Mickiewicz University Poznań Poland; ^9^ Organoid Platform, Berlin Institute for Medical Systems Biology (BIMSB), Max Delbrück Center for Molecular Medicine (MDC) Berlin Germany; ^10^ Department of Neurosurgery Józef Struś Hospital Poznań Poland; ^11^ Intraoperative Imaging Unit, Department of Neurosurgery and Neurotraumatology Poznań University of Medical Sciences Poznań Poland; ^12^ Department of Neurosurgery and Neurotraumatology Heliodor Święcicki Clinical Hospital Poznań Poland

**Keywords:** circRNA, GBM, RBP, RNA‐seq

## Abstract

Glioblastoma (GBM) is the most aggressive and lethal type of glioma, characterized by aberrant expression of noncoding RNAs including circular RNAs (circRNAs). CircRNAs may impact cellular processes by interacting with other molecules—like RNA‐binding proteins (RBPs). The diagnostic value of circRNA and circRNA/RBP complexes is still largely unknown. To explore circRNA and RBP transcript expression in GBM, we performed and further analyzed RNA‐seq data from GBM patients' primary and recurrent tumor samples. We identified circRNAs differentially expressed in primary tumors, the circRNA progression markers in recurrent GBM samples, and the expression profile of RBP genes. Furthermore, we demonstrated the clinical potential of circRNAs and RBPs in GBM and proposed them as stratification markers in *de novo* assembled tumor subtypes. Additionally, we experimentally validated the subcellular localization of select circRNAs and their interactions with FUS. Subsequently, we showed that circARID1A may play a role in promoting GBM cell proliferation. Overall, we described circRNA‐RBP interactions that could play a regulatory role in gliomagenesis and GBM progression and provided a list of molecular players in GBM for further extensive studies.

## Graphical abstract

CircRNAs are differentially expressed in glioblastoma primary tumors and might serve as therapeutic targets and diagnostic markers. The investigation of circRNA and RNA‐binding proteins (RBPs) interactions shows that distinct RBPs play a role in circRNA biogenesis and function. Furthermore, circRNAs and RBPs demonstrated clinical potential and RBP expression led to the new subtype classification, enhancing patient stratification.

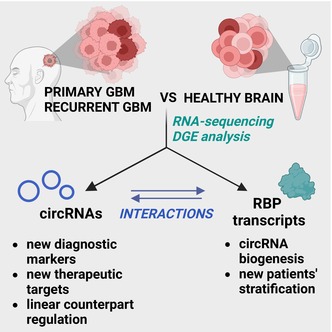

**Abbreviations**
circRNA: circular RNA
CLIP: cross‐linking and immunoprecipitation
CLR: circular‐to‐linear ratio
DCE: differential circRNA expression
DGE: differential gene expression
DMEM: Dulbecco's modified Eagle's medium
EMEM: Eagle's Minimal Essential Medium
FBS: fetal bovine serum
GBM: Glioblastoma
GBM‐PRM: primary glioblastoma
GBM‐REC: recurrent glioblastoma
HB: healthy brain
IDH1, IDH2: isocitrate dehydrogenase
miRNA: microRNA
ncRNA: noncoding RNA
OVS: overall survival
RBP: RNA‐binding protein
RNA‐seq: RNA sequencing
RT‐qPCR: reverse transcription‐quantitative polymerase chain reaction
siRNA: small interference RNA
TCGA: The Cancer Genome Atlas

## 1 Introduction

Gliomas are brain neoplasms originating from the glia and constitute most of all primary tumors in the central nervous system. They are classified into four grade groups, based on histological, genetic, and prognostic features. Low‐grade gliomas (I and II grades) exhibit minor metastasis and recurrence potential, while high‐grade gliomas (III and IV grades) present significant recurrence and progression prognosis [1]. Glioblastoma (GBM)—an IV‐grade glioma is the most lethal, aggressive, and malignant among brain tumors in adults, with a median survival of 14.6 months post‐treatment [2]. Current therapies, which are not curative, include radiotherapy and temozolomide‐based chemotherapy [3]. Due to the lack of effective treatments, high internal molecular heterogeneity among GBM tissues, and high rate of recurrence, there is an urgent need to understand the molecular basis of GBM better and improve patient stratification, defining signatures useful as potential diagnostic and prognostic markers and most importantly to identify new therapeutic targets.

The most common molecular stratification parameter of GBM is the presence of mutations in isocitrate dehydrogenase—*IDH1* and *IDH2* genes. These mutations are most frequent in secondary GBM and predict a favorable disease outcome with prolonged median survival [4, 5]. An additional molecular classification of GBM comprises of four submolecular groups: proneural, classical, mesenchymal, and neural which are distinguished by specific gene expression patterns, mutations and associated with different tumor aggressiveness and prognosis for treatment response [6].

In recent years, noncoding RNAs (ncRNAs) and particularly circular RNAs (circRNAs) have gained attention as key players in cancer development and potential therapeutic targets. CircRNAs are single‐stranded RNA molecules produced by ‘back‐splicing’, which joins a downstream 5′ donor splice site and an upstream 3′ acceptor splice site [7]. Due to their intrinsic resistance to exonuclease cleavage, circRNAs have a longer half‐life in comparison to their linear counterparts making them promising cancer biomarkers [8, 9, 10, 11, 12, 13, 14, 15].

Recent studies have highlighted the role of RNA‐binding proteins (RBPs) in the biogenesis and maintenance of circRNAs, influencing the transcriptome's molecular balance [16, 17]. CircRNAs can also act as microRNA (miRNA) and RBPs sponges potentially impacting gene expression and cellular pathways significantly [12, 18, 19, 20, 21, 22, 23, 24]. Interactions with miRNAs represent a particularly intriguing and extensively described function of circRNAs as they can modify miRNA functionality by regulating their bioavailability. CircRNAs contain binding sites for specific miRNAs, acting as molecular sponges, thereby hindering miRNAs from binding to their target mRNAs and leading to increased expression levels of the protein. A single circRNA molecule can bind to several different miRNAs simultaneously and/or possess multiple binding sites for the same miRNA. Consequently, they can regulate the balance between miRNAs and their targets by modulating their biological accessibility within cells [23, 25]. The interactions between circRNAs and RBPs rely on binding motifs and contextual features, such as secondary structure, flanking nucleotide composition, or short nonsequential motifs [26]. Apart from the RBPs binding motifs, the biogenesis of circRNAs can also be facilitated by the presence of the complementary sequences in both flanking regions, like *Alu* elements [27]. Understanding the nature and function of such interactions is crucial for understanding GBM, including tumor biology, progression, treatment response, and survival rates [28, 29, 30].

In this study, we provide the comprehensive atlas of circRNAs and RBP genes differentially expressed in GBM. By utilizing deep total RNA sequencing (RNA‐seq), we established the expression profile of circRNAs and extensively studied their interaction with RBPs, including the potential impact on circRNA formation. First, we identified the differentially expressed circRNA in primary GBM patients' samples (GBM‐PRM) and in the recurrent GBM samples (GBM‐REC). We further experimentally validated the expression of selected circRNAs and their unique backsplice junction sequences and structures. Furthermore, we confirmed their subcellular localization, and using the siRNA‐based approach, we performed the initial functional study indicating the possible function of circARID1A in promoting the proliferation of GBM cells. On top of that, using the same dataset, we analyzed the expression profiles of RBP genes in GBM. This investigation into circRNAs and RBP transcripts revealed potential binding sites within circRNA sequences and identified RBPs that may play a role in circRNA biogenesis and function.

The performed co‐immunoprecipitation, identified FUS as the interactor of the selected circRNAs, confirming our *in silico* predictions. Further analysis correlated circRNA and RBP expression with established GBM subtypes, leading to a new subtype classification that enhances patient stratification and identification of prognostic markers.

Our global analysis pointed circRNAs significantly deregulated in GBM, offering new insights into potential diagnostic and therapeutic targets.

## 2 Materials and methods

### 2.1 Patients' sample collection

Tumor tissues (*n* = 26; Table S1) were collected from GBM patients up to one hour after tumor excision. The material was obtained from the Department and Clinic of Neurosurgery and Neurotraumatology of the University of Medical Sciences in Poznan (Poland) and from the Department of Neurosurgery of Multidisciplinary City Hospital in Poznan (Poland). Prior to the surgery, the approval by the Bioethics Council of the Poznan University of Medical Science (Nr. 46/13) and individuals signed an informed written consent form had been obtained. Four commercial samples (purchased from Ambion, Austin, TX, USA; Clontech, Mountain View, CA, USA; Takara Bio, Shiga, JP) of pooled human brain altogether containing total RNA from 52 individuals were used as healthy brain controls (HB) (Table S2). The study methodologies conformed to the standards set by the Declaration of Helsinki.

### 2.2 RNA extraction and quality check

Total RNA extraction from GBM patient tissue was performed using TRIzol reagent (Invitrogen, Waltham, MA, USA) according to the manufacturer's protocol. RNA samples have been subjected to DNase I treatment using a ready‐to‐use DNA‐free™ DNA Removal Kit reagents following the manufacturer's protocol (Ambion, Austin, TX, USA). The quality of purified RNA was measured by NanoDrop 2000 spectrophotometer (Thermo Fisher Scientific, Waltham, MA, USA) followed by agarose gel at a concentration of 1% electrophoresis. RNA integrity was verified on Agilent Bioanalyzer 2100 (Agilent Technologies, Santa Clara, CA, USA). RNA samples with RNA Integrity Number (RIN) at least 7.4 were used for library preparation and RNA‐seq analysis (Figs S1, S2).

### 2.3 Library preparation and RNA sequencing

About 300 ng of total RNA was ribosomal RNA‐depleted using RNase H [23]. Ribosomal RNA‐depleted libraries were constructed using Illumina's TruSeq Total RNA Library Prep Kit (San Diego, CA, USA). Adaptor ligations were performed according to manufacturer's instructions and cDNA fragments were amplified in RT‐qPCR for 8–15 cycles. After the purification with AMPure XP beads, the DNA concentration was measured with Qubit (Thermo Fisher Scientific, Waltham, MA, USA) and the fragments length were defined with Screen Tape Assay Agilent D1000 (Agilent Technologies, Santa Clara, CA, USA), 4200 Tape Station System (Agilent Technologies, Santa Clara, CA, USA). RNA sequencing was performed using Illumina Hi‐seq 4000 (Illumina, San Diego, CA, USA), average of ~73 million of 150 paired‐end reads per sample.

### 2.4 circRNAs identification

Quality control of raw sequencing reads was done with FastQC (https://www.bioinformatics.babraham.ac.uk/projects/fastqc/). Next, the adapters were removed using trimmomatic [31], version 0.38 with the following parameters ILLUMINACLIP:2:30:10 SLIDINGWINDOW:10:25 MINLEN:35. After adapter trimming, a second quality check with FastQC was performed. A genomic index was created for GRCh37 human genome obtained from Gencode using Burrows‐Wheeler aligner [32], version 0.7.17 (bwa–bwtsw). Then, reads were mapped with the following parameters: bwa‐mem–T 19. CircRNAs were detected from alignment files with CIRI [33], version 2.0.6 using default parameters. CircRNAs annotation and differential expression analysis were performed using circMeta R package [34]. The edgeR [35] method was used for differential expression analysis. Heatmaps were prepared using the pheatmap R package.

Data were visualized using the ggplot2 R package.

### 2.5 RBPs identification

To identify RBPs differentially expressed between HB and GBM samples, trimmed reads were mapped to the human genome (GRCh37 v30 from Gencode) using Burrows‐Wheeler aligner [32], version 0.7.17, followed by mapping sequencing reads to genomic features using featureCounts function from Rsubread package with useMetaFeatures option and without counting multimapping and multi‐overlapping reads. Differential gene expression analysis was performed using edgeR glmQLFTest [35]. RBPs were selected from differentially expressed genes based on the human RBP list, obtained from Gerstberger *et al*. [36].

### 2.6 circRNAs‐RBPs interactions

To identify putative RBP‐binding sites in differentially expressed circRNA, first, circRNA sequences were extracted using FcircSEC [37] followed by k‐mer enrichment analysis between differentially expressed and nondifferentially expressed circRNAs. K‐mer enrichment analysis was performed with *k* = 6, using kmer_compare function from the FeatureReachR package. Then, 6‐mers enriched in differentially expressed circRNAs were mapped to the RBPs binding sites determined either by eCLIP [38] or by RNA bind and Seq experiments [26], using estimate_motif_from_kmer function from FeatureReachR and CISBPRNA_hs or RBNS position weighted matrices lists, respectively. Significantly enriched RBP‐binding motifs (adjusted *P*‐value <0.1) were visualized using ggseqlogo R package [39]. Analysis was then repeated for circRNA flanking regions (1000 nt flanks).

Correlation of expression of RBPs and differentially expressed circRNA was estimated using cor.test function from R with Spearman rank correlation option. The Upset plot was prepared using the UpSetR package [40].

### 2.7 GBM subtypes analysis based on circRNAs

Raw sequencing data were subjected to the quality check using FastQC tool. The adaptors were trimmed and the reads were filtered to eliminate the low‐sequencing‐quality bases using trimmomatic [31]. Furthermore, the RNA‐seq reads were mapped to the human reference transcriptome (ENSEMBLE V.102) to quantify the expression level of the transcripts. The transcript‐level estimates were summarized and associated with the gene IDs for gene‐level analysis using tximport [41]. For the sample categorization, we used genes indicated in TCGA GBM dataset and other genes that have been found to be significant for GBM molecular subtyping including *SLC12A5*, *SYT1*, *GABRA1*, *NEFL*, *CDKN1A*, *NF1*, *MET*, *PDGFRA*, *BOP1*, and *ILR4* were included. CircRNAs differentially expressed within the subtypes were identified using circMeta R package [34] with edgeR [35] method for differential circRNAs expression (DCE).

### 2.8 GBM sample stratification based on RBPs expression

GBM sample stratification according to RBPs expression changes was performed using the CircIMPACT workflow [42]. At first, we subset RBPs able to discretize patients into two clusters according to their expression with no significant association with GBM molecular subtypes (χ^2^ test, *P*‐value >0.01). We clustered patients in novel subgroups linked by common RBPs pattern expression. The unsupervised clustering was performed using the k‐means algorithm with Euclidean distance and automatic selection of the optimal number of k clusters of patients using the silhouette index. Finally, we tested which RBPs reach significant estimates of the expression changes between previously defined clusters (one‐way ANOVA test *P*‐values ≤0.05). After correction for multiple tests, the RBPs with significant expression variation were used to compose the list of discriminant RBPs for the corresponding sample clustering.

### 2.9 Survival analysis

Survival analysis was performed on the GBM dataset from TCGA. Log_2_ transformed HTseq counts were downloaded using UCSC Xena tool [43]. Log_2_ transformation was reversed, and raw reads were normalized using TMM method from edgeR R library [35]. Then, differentially expressed RBPs were selected (listed in Table S3) and for each RBP quartile normalization was applied. Samples belonging to Q1 were marked as ‘low’, Q2 and Q3—‘medium’ and Q4—‘high’. Survival analysis was performed using survival and survminer R libraries between samples with ‘low’ and ‘high’ expression levels. Log‐rank (Mantel–Haenszel) test was used to assess statistical significance (*P*‐value <0.05). Data were visualized using ggsurvplot function from survminer library.

### 2.10 Cell culture

Human GBM cell lines U‐118 MG (Cellosaurus Database Accession: CVCL_0633) and U‐251 MG (Cellosaurus Database Accession: CVCL_0021) were purchased from American Type Culture Collection (ATCC, Manassas, VA, USA), and after two passages, they were aliquoted and stored at −80 °C until used. Cells used in this study were between passages 5–15, after the cells were replaced with a freshly thawed sample. Cells were maintained in the medium recommended by the manufacturer—Eagle's Minimal Essential Medium (EMEM, Corning, NY, USA) (U‐251 MG) or Dulbecco's modified Eagle's medium (DMEM, ATCC, Manassas, VA, USA) (U‐118 MG), supplemented with 1% penicillin–streptomycin antibiotic (Sigma‐Aldrich, St. Louis, MO, USA) and 10% fetal bovine serum (FBS, Sigma‐Aldrich, St. Louis, MO, USA). Cells were incubated in the conditions for optimal growth and maintenance: 37 °C, 95% humidity, and 5% CO_2_ concentration. They were routinely tested for mycoplasma contamination using LookOut^®^ Mycoplasma PCR Detection Kit (Sigma‐Aldrich, St. Louis, MO, USA). Additionally, the authenticity of the cell lines was verified by examining their DNA‐short tandem repeat (STR) profiles by using the AmpFLSTR™ Identifiler™ Plus PCR Amplification Kit (Thermo Fisher Scientific, Waltham, MA, USA).

### 2.11 Subcellular fractionation

Cytoplasmic and nuclear fractions were separated according to the protocol described by Conrad and Ørom [44]. Briefly, a total of 5 × 10^6^ U‐118 MG and U‐251 MG cells were washed in PBS and incubated with trypsin solution at 37 °C for 5 min. The trypsinization reaction was stopped by adding 10 mL cold EMEM or DMEM. The cell suspension was transferred into a Falcon tube and centrifuged at 200 ***g*** for 5 min. Then, the cell pellet was resuspended twice in PBS and centrifuged at 200 ***g*** for 5 min and 2 min. The cell pellet was subjected to the lysis with Igepal lysis buffer comprised of 10 mm Tris pH 7.4, 150 mm NaCl, 0.15% Igepal CA‐360, and 20 U·mL^−1^ of Protector RNase Inhibitor (Merck, Darmstadt, DE) and incubated for 5 min. To obtain separated cytoplasmic and nuclear fractions, the cell lysate was overlaid on top of the sucrose buffer comprised of 10 mm Tris pH 7.4, 150 mm, 24% sucrose, and 20 U·mL^−1^ of Protector RNase Inhibitor (Merck, Darmstadt, DE) and centrifuged at 3500 ***g*** for 10 min. Then, the supernatant containing cytoplasmic fraction was centrifuged at 14 000 ***g*** for 1 min and the pellet containing nuclei was rinsed with 1 mL ice‐cold PBS‐EDTA and centrifuged at 3500 ***g*** for 5 s. All the centrifugation was carried out at 4 °C, and each incubation was performed on ice. Furthermore, the RNA was extracted from both fractions using 1 mL of TRIzol reagent per each 200 μL of cytoplasmic extract and per nuclear pellet.

### 2.12 Transfection with siRNA

2.5 × 10^5^ of U‐118 MG and U‐251 MG GBM cells at 70–80% confluency was transfected with siRNAs in a final concentration of 50 nm, utilizing siPORT™ Amine as a transfection agent (Thermo Fisher Scientific, Waltham, MA, USA). A nonspecific scrambled siRNA was used as a control in all transfection experiments. Transfected cells were subjected to downstream experiments after 24 and 48 h. SiRNAs used in the study were purchased in FUTUREsynthesis (Poznan, PL), and their sequences are listed in Table S4.

### 2.13 Real‐time proliferation

Real‐time cell proliferation monitoring was performed in the xCELLigence^®^ system using the RTCA DP apparatus (ACEA Biosciences, San Diego, CA, USA). The experiment was carried out on 16‐well E‐Plates (ACEA Biosciences, San Diego, CA, USA), whose well bottoms are covered with gold microelectrodes. The test was started by measuring the background impedance of supplemented medium by placing them in the RTCA DP apparatus (ACEA Biosciences, San Diego, CA, USA) and making the first measurement. Then, 5 × 10^3^ U‐118 MG or U‐251 MG GBM cells were seeded on the same plates and incubated under optimal growth conditions. After 24 h, cells were transfected with 50 nm of siRNA specific for circARID1A and circPLOD2 or scrambled control. From that moment on, until the end of the experiment, the system performed impedance measurements at 15‐min intervals for 48 h. The results are presented by the cell index unit. The normalization time point corresponds to the moment of transfection.

### 2.14 Cross‐linking and immunoprecipitation (CLIP)

GBM cells were cultured on 150 mm diameter dishes (minimum 90% confluency) and washed with PBS buffer. Then, 10 mL of cold PBS buffer was added and transferred to ice. The cells were cross‐linked on ice using UV radiation at a wavelength of 254 nm and an intensity of 0.4 J·cm^−2^. After this procedure, the PBS buffer was removed and 1 mL of RIP buffer comprised of 20 mm Tris pH 8.0, 100 mm NaCl, 0.5 mm EDTA, 0.5% NP40, and 0.1% SDS was added to the cells, transferred to Eppendorf tubes, and incubated for 20 min. on ice. The cell mixture was passed through a 21G needle 7 times using a 2 mL syringe and centrifuged for 10 min. at 13000 RPM at 4 °C. After centrifugation, the supernatant was transferred to a new Eppendorf tube and the pellet was discarded. The protein concentration was then assessed using the BCA method (Thermo Fisher Scientific, Waltham, MA, USA). Then, 1 mg of protein was mixed in a 2 mL Eppendorf tube with 1 μg of antibodies against FUS (Invitrogen, Waltham, MA, USA, PA5‐96477) or IgG (Abcam, Cambridge, UK, ab2410). Additionally, an ‘input’ was prepared in a separate tube, constituting 10% of the volume of the protein used. Then, the samples were incubated on a rotor at 4 °C for 2 h. Next, 30 μL of Dynabeads™ Protein G for Immunoprecipitation (Thermo Fisher Scientific, Waltham, MA, USA) were prepared for the sample by washing them twice with an equivalent volume of RIP buffer and finally dissolving them in 30 μL of RIP buffer. The magnetic beads were added to the previously prepared samples and incubated on a rotor at 4 °C for 1 h. After incubation, the supernatant was separated using a magnetic separator and removed. The magnetic beads with bound protein–RNA complexes were washed three times with 800 μL of Wash Buffer comprised of 50 mm Tris pH 7.4, 100 mm NaCl, 1 mm MgCl_2_, 0.05% NP40 and then three times with 800 μL of High‐salt Wash Buffer comprised of 50 mm Tris pH 7.4, 500 mm NaCl, 1 mm MgCl_2_, 0.05% NP40. During each wash, the samples were mixed on the rotor for 2 min at room temperature. In the last step, the samples were washed with RIP buffer and mixed on the rotor for 2 min at room temperature. Then, each sample was dissolved in 60 μL of RIP buffer. 1/3 of the volume was used for western blotting and 2/3 of the volume for RNA isolation. To extract the RNA, 5 μL of proteinase K was added and adjusted to 100 μL with proteinase K buffer comprised of 100 mm NaCl, 10 mm Tris pH 7.4, 0,5% SDS, 1 mm EDTA, followed by incubation for 1 h at 70 °C with shaking at 300 RPM. Subsequently, 700 μL of TRIzol was added and the RNA extraction was performed according to the protocol described in ‘4.2 RNA extraction and quality check’ with overnight incubation at −80 °C after the addition of isopropanol.

### 2.15 Western blotting

Samples from the CLIP experiment with 5 μL of 4 × Protein Loading Buffer (EURx, Gdansk, PL) were added. Magnetic beads were separated using a magnetic separator, and the supernatant was loaded in SDS/PAGE sample buffer and separated using 12% SDS/PAGE. Proteins were transferred to the PVDF membrane by wet blotting and then membranes were blocked for 1 h at room temperature with 5% nonfat milk in PBS 0.05% containing Tween‐20 (PBS‐T). Following membranes were incubated with primary FUS antibody (Invitrogen, Waltham, MA, USA, PA5‐96477) or HPRT antibody (Abcam, Cambridge, UK, ab10479) at 1:1000 dilution overnight at 4 °C. After incubation, blots were washed with PBS‐T 3 times and incubated with VeriBlot antibody (Abcam, Cambridge, UK, AB131366) at 1:100 dilution for 1 h. Protein bands were visualized using Pierce™ ECL Western Blotting Substrate (Thermo Fisher Scientific, Waltham, MA, USA) on Alliance Q9 (Uvitec, Cambridge, UK).

### 2.16 Reverse transcription and quantitative RT‐qPCR

The reverse transcription reaction was proceeded using the Transcriptor High Fidelity cDNA Synthesis Kit (Roche, Basel, CHE) according to the manufacturer's protocol. 500 ng of total RNA extracted from patient‐derived tissues was used. The real‐time qPCR reaction was performed using the CFX Connect Real‐Time PCR Detection System (Bio‐Rad, Hercules, CA, USA) on the 96‐well plates recommended by the manufacturer. Each sample was run in three technical replicates. RT‐qPCR analysis was performed with LightCycler^®^ 480 SYBR Green I Master (Roche, Basel, CHE) and primers designed using the Primer‐BLAST tool [45] and purchased from Thermo Fisher Scientific (Waltham, MA, USA). Primers' sequences are listed in the Table S5. The expression level of the studied genes was calculated in CFX maestro 2.0 software. Hypoxanthine phosphoribosyltransferase (*HPRT*) was used as an endogenous control for calculating the expression levels of selected genes. As control, commercially available RNA from HB tissues was used (Table S2).

### 2.17 RNase R treatment

2 μg of total RNA were treated with 4 U of RNase R (Lucigen, Middleton, WI, USA) at 37 °C for 5 or 10 min in case of downregulated and at 37 °C for 30 min for upregulated circRNAs followed by 20 min at 65 °C. Next, total RNA was purified using NucAway Spin Columns (Invitrogen, Waltham, MA, USA) and reverse transcribed with the Transcriptor High Fidelity cDNA Synthesis Kit (Roche, Basel, CHE) according to the manufacturer's protocol. Then, PCR was performed with 35 cycles (15 s denaturation at 95 °C followed by 30s at the optimum annealing temperature for each pair of primers (Table S5) and 20s extension) with a prior 3‐min denaturation. The depletion of linear RNA was confirmed by running the PCR products on agarose gel at a concentration of 1.2%.

### 2.18 Statistical analysis of the results

Each experiment was performed in three biological replicates including three technical replicates each. Statistical significance of the differences observed in experimental data was calculated in R, using *t*‐test (stat_compare_means function) if not indicated differently on the figure legend. *P*‐values are presented in plots.

## 3 Results

### 3.1 circRNAs and RBPs transcripts are differentially expressed in GBM

#### 3.1.1 Expression profile of circRNA in GBM

**Fig. 1:**
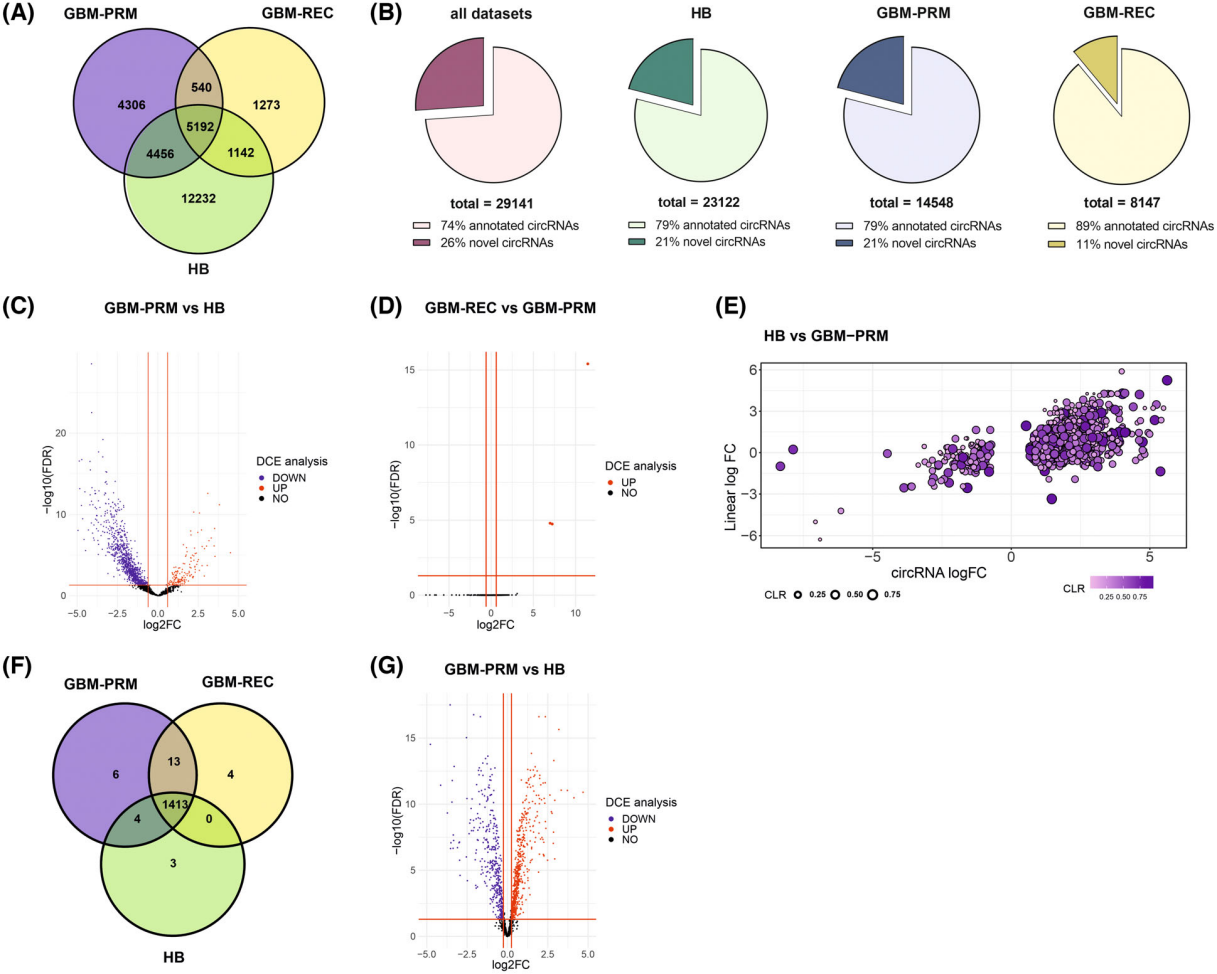
Overview of the identified circRNAs and RBPs in GBM. (A) Venn diagram illustrates an overlap between circular RNAs (circRNAs) expressed in analyzed primary glioblastoma (GBM‐PRM) (*n* = 23), recurrent glioblastoma (GBM‐REC) (*n* = 3) tissues, and healthy brain (HB) control (*n* = 52). (B) Distribution of novel and annotated circRNAs in selected samples. (C) Volcano plot of differentially expressed circRNAs in GBM‐PRM (*n* = 23) vs HB (*n* = 52). The blue and red dots indicate downregulated and upregulated circRNAs, respectively. (D) Volcano plot of differentially expressed circRNAs in GBM‐REC (*n* = 3) vs GBM‐PRM (*n* = 23). The color code is the same as in C. (E) Scatter plot presenting the expression changes of circRNA (x‐axis) and the corresponding mRNA (y‐axis). Each dot color represents a circular‐to‐linear ratio (CLR) for a given circRNA‐mRNA pair. (F) Venn diagram illustrates an overlap between RBP genes expressed in analyzed GBM‐PRM (*n* = 23), GBM‐REC (*n* = 3) tissues, and HB control (*n* = 52). (G) Volcano plot of differentially expressed RNA‐binding protein (RBP) genes in GBM‐PRM vs HB. The color code is the same as in C.

To define circRNA and RBP gene expression profiles in primary (GBM‐PRM, *n* = 23) and recurrent GBM (GBM‐REC, *n* = 3) (Table S1) in comparison to the healthy brain control altogether containing total RNA from 52 individuals (HB) (Table S2), total RNA sequencing was performed. We chose total RNA‐seq to profile both circRNA and messenger RNA transcripts simultaneously. We identified a total number of 29 141 circRNAs in all samples (Fig. 1A) including more than 7600 previously unannotated (Fig. 1B). The majority of identified circRNA were derived from exons (Fig. S3A) and their length was similar among all analyzed samples with the median equal: 740, 631, and 555 nucleotides for HB, GBM‐PRM, and GBM‐REC, respectively (Fig. S3B). The circRNA transcripts were distributed within all human chromosomes, including chromosomes 1 and 2 circRNAs (Fig. S4). CircRNA expression profiles showed clear discrimination between GBM‐PRM, GBM‐REC, and HB samples (Fig. S5). The differential circRNA expression (DCE) analysis revealed 1270 circRNAs differentially expressed between GBM‐PRM and HB tissues with a log_2_ fold change in the range between −5.6 and 8.9 (adjusted *P*‐value <0.05) where 1132 of circRNAs were downregulated and only 138 were upregulated (Fig. 1C). To search for circRNAs potentially involved in GBM progression, we performed further DCE analysis between GBM‐PRM and GBM‐REC where we detected 3 differentially expressed circRNAs (Fig. 1D). To determine the relationship between deregulated circRNAs and their linear counterparts, we calculated the circular‐to‐linear ratio (CLR) based on the formula [circ/(circ + lin)]. A CLR value >0.5 indicates that the number of circRNA molecules is higher than the corresponding mRNA, while a CLR value <0.5 indicates the opposite. Interestingly, the obtained results showed that in most cases, the number of circRNAs was higher than the number of their parental linear counterparts (CLR >0.5). Moreover, both circular and linear molecules were frequently upregulated, indicating a positive correlation between their expression levels (Fig. 1E, upper right quadrant of the figure).

#### 3.1.2 Expression profile of genes encoding RBPs in GBM

As mentioned previously, RBPs play a pivotal role in circRNA biogenesis, thus they might be involved in regulating the balance between the expression of circRNAs and their host genes. Therefore, we performed the analysis of the expression profile of RBP genes based on RNA‐seq obtained from the same dataset. We identified 1420 RBP genes expressed in HB, 1436 in GBM‐PRM, and 1430 in GBM‐REC samples (Fig. 1F). Differential gene expression (DGE) analysis of RBP genes resulted in the finding that 817 from 1542 known human RBP [36] are characterized by the significantly altered expression in GBM‐PRM (469 up‐ and 348 downregulated) with a log_2_ fold change in the range between −4.8 and 4.7 (Fig. 1G; Fig. S6).

### 3.2 Characteristics and validation of circRNAs dysregulated in GBM‐PRM and GBM‐REC

The DCE analysis revealed a list of differentially expressed circRNA among GBM‐PRM and HB samples which may suggest their role in processes related to the development of this tumor type. The potential progression markers were additionally identified by the comparative expression analysis between GBM‐PRM and GBM‐REC samples. Eleven circRNAs were selected for further validation based on the list of mostly downregulated and upregulated molecules in GBM.

Based on the analysis of GBM‐PRM vs HB control, we selected four upregulated circRNAs (circARID1A, circGUSBP1, circPLOD2, and circVCAN) and four downregulated ones (circCADPS2, circEPB41L5, circUNC13C, and circUSP45). The DCE analysis between GBM‐REC and GBM‐PRM allowed us to distinguish 3 circRNAs specifically upregulated in recurrent samples: circEGFR, and two new, not previously annotated, circRNAs coming from *HLA‐B* gene, and intergenic circRNA originating from chromosome 6p22.1 named for convenience as circInter6p22 (Table S6).

**Fig. 2:**
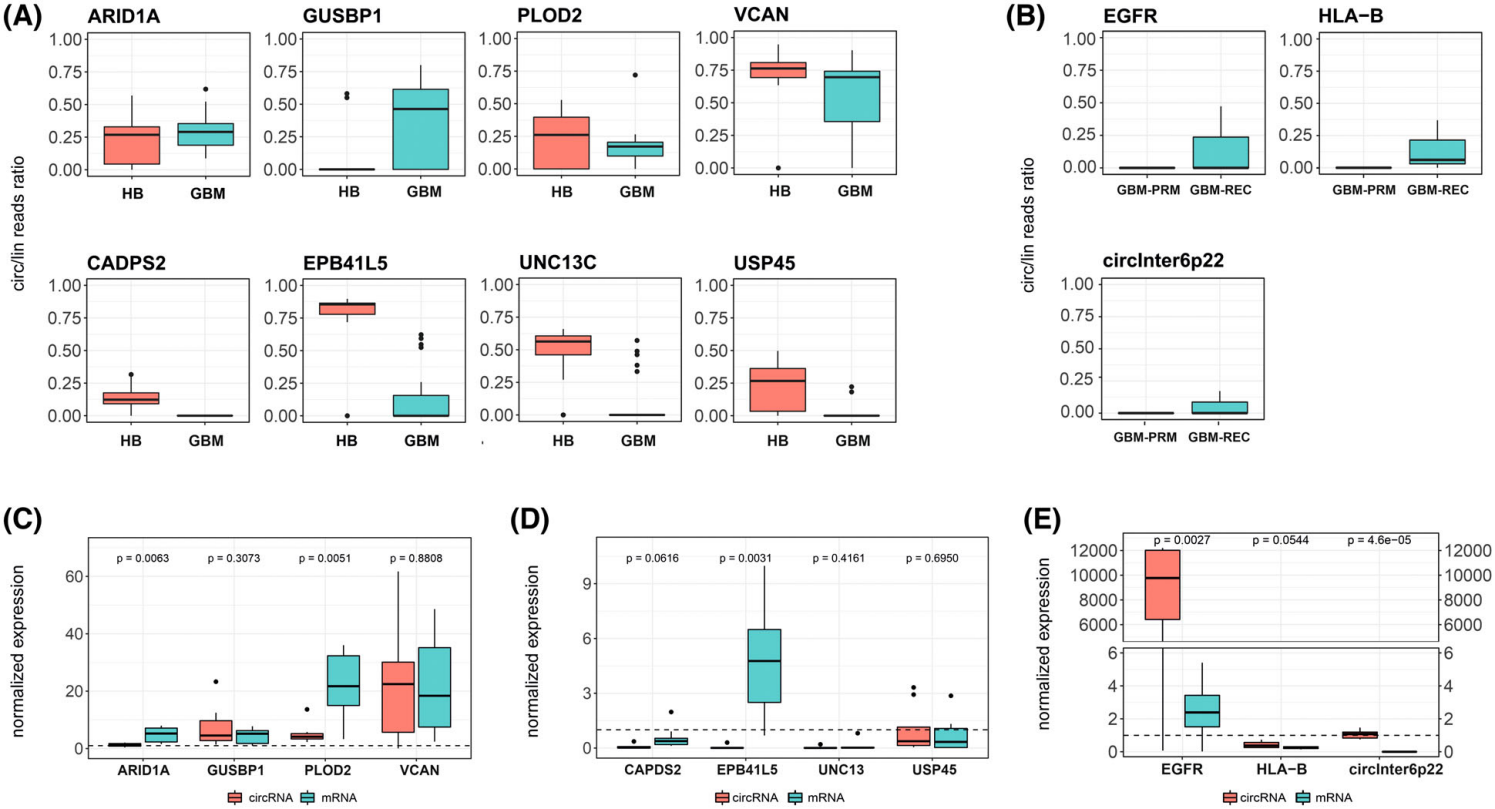
Expression of selected circRNA dysregulated in GBM and their corresponding mRNAs. (A) Boxplots illustrating the ratio of circular to linear RNA expression for selected transcripts dysregulated in primary glioblastoma (GBM‐PRM) (*n* = 23) in comparison to healthy brain (HB) (*n* = 52). (B) Boxplots illustrating the ratio of circular to linear RNA expression levels for selected transcripts dysregulated in recurrent glioblastoma (GBM‐REC) (*n* = 3) in comparison to GBM‐PRM (*n* = 23). (C–E) Boxplots illustrating circular and linear RNA expression levels from RT‐qPCR for selected candidates upregulated in GBM‐PRM (*n* = 8) (C), downregulated in GBM‐PRM (D), and upregulated in GBM‐REC (*n* = 3) (E). Expression levels are normalized to HB (*n* = 52) (C, D) and GBM‐PRM (E), indicated as dashed horizontal lines in all 3 panels.

We have firstly performed a comparison of the circular to linear read ratio of selected circRNAs based on RNA‐seq data, which showed that in almost all cases the circular transcript is less abundant than the linear form in GBM, with the exception of VCAN circular transcript which was more expressed than its linear counterpart. Interestingly, we also perceived that the relation between circular and linear transcripts might differ between HB and GBM. For instance, circEPB41L5 is more abundant than its parental transcript in healthy brain, but this interplay is reversed in GBM where the linear form is more abundantly expressed (Fig. 2A,B).

In order to confirm the circular structure of the identified molecules, the RNase R treatment was performed (Fig. S7). All of the selected circRNAs showed resistance to exonuclease treatment and higher stability than their cognate mRNA. Subsequently, we used 8 GBM‐PRM samples that were utilized in RNA‐seq, to validate circRNAs expression using RT‐qPCR (Table S1).

Among upregulated circRNA candidates, VCAN showed the highest expression changes (Fig. 2C). Regarding downregulated circRNAs, we observed simultaneous downregulation of linear transcripts with one exception—circEPB41L5 in which the linear form was upregulated (Fig. 2D). RT‐qPCR validation was also performed for circRNAs upregulated in GBM‐REC in comparison to GBM‐PRM. We noted the exceptionally high level of circEGFR upregulation reaching ~12 000‐fold change and higher relative upregulation of circRNA in comparison to their linear counterparts (Fig. 2E). Generally, RT‐qPCR supported expression changes observed in RNA‐seq (Fig. S8A,B). We also observed a significant positive correlation for UNC13C, VCAN (GBM‐PRM) and EGFR, HLA‐B (GBM‐REC) in RT‐qPCR data (Fig. S8C,D).

### 3.3 CircARID1A knockdown leads to decreased proliferation rate of GBM cells

In order to functionally validate the selected differentially expressed circRNAs, we subjected the upregulated ones, namely: circARID1A, circPLOD2, circGUSBP1, and circVCAN to further studies.

**Fig. 3:**
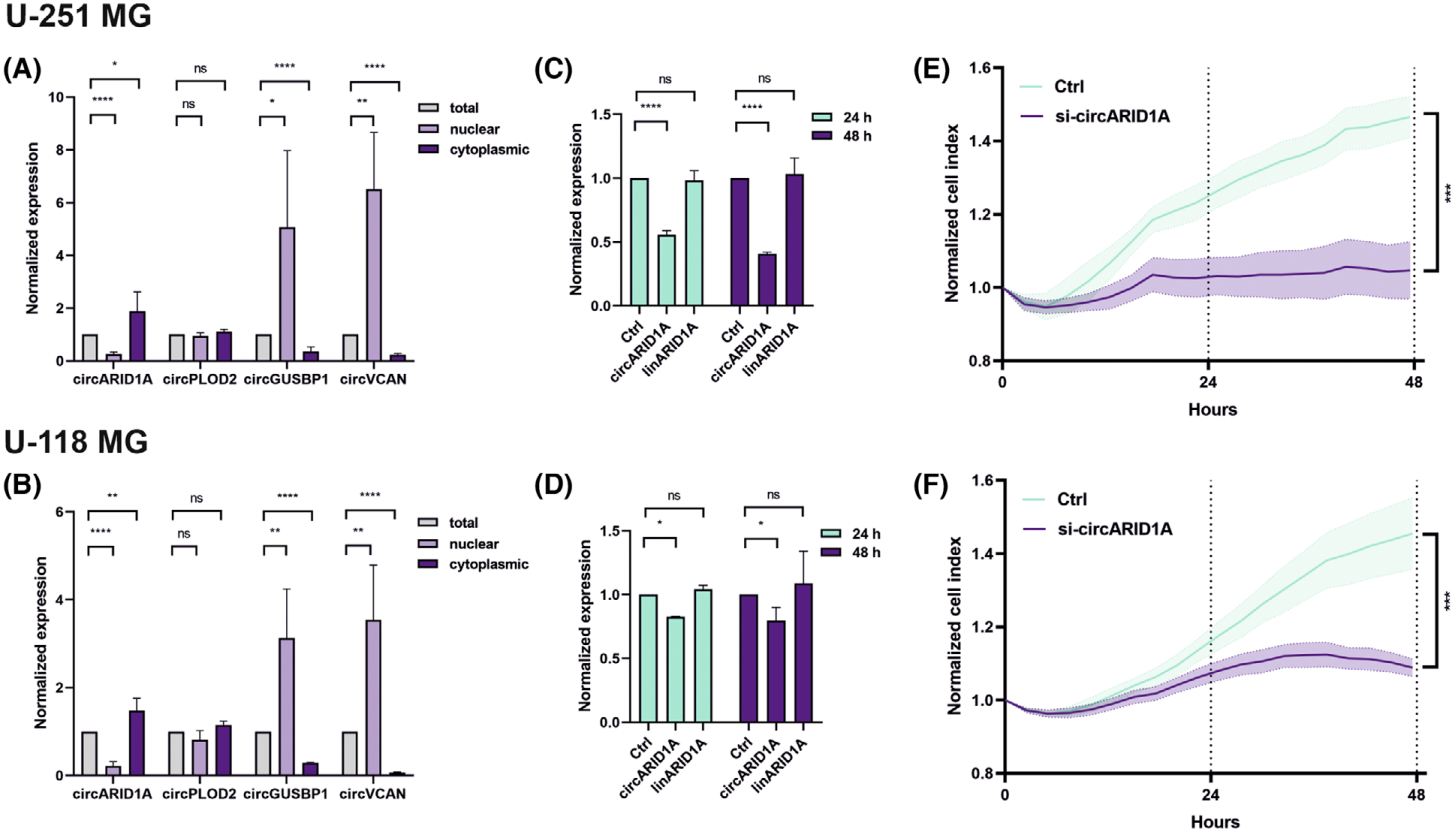
CircARID1A knockdown leads to decreased proliferation rate of GBM cells. (A, B) Bar plots showing the subcellular localization of selected upregulated circular RNAs (circRNAs) in U‐251 MG (A) and U‐118 MG (B) glioblastoma (GBM) cell lines. Total: whole‐cell RNA, nuclear: nuclear fraction, cytoplasmic: cytoplasmic fraction. (C, D) The normalized expression level of circARID1A and linARID1A after knockdown of circARID1A in U‐251 MG (C) and U‐118 MG (D) GBM cell lines after 24 and 48 h. The efficiency of the downregulation was established by qRT‐PCR analysis and scrambled siRNA served as a control (Ctrl). (E, F) Proliferation rates after circARID1A knockdown in U‐251 MG (E) and U‐118 MG (F) GBM cell line. Results are presented as mean values ± SD of 3 biologically independent replicates, normalized to reference HPRT gene and compared to control. Statistical significance was calculated using one‐way ANOVA test: ns, not statistically significant; * for *P* < 0.05; ** for *P* < 0.01; *** for *P* < 0.001; **** for *P* < 0.0001.

First, we performed subcellular fractionation which revealed, that circARID1A is predominantly localized in the cytoplasm, circGUSBP1 and circVCAN in the nucleus, while circPLOD2 is present in both cellular compartments (Fig. 3A,B).

To verify the potential role of the identified circRNAs, we applied siRNA treatment in U‐118 MG and U‐251 MG GBM cell lines for circARID1A and circPLOD2 since these molecules were only accessible due to their cytoplasmatic localization. For circARID1A, we observed ~45% and ~60% after 24 and 48 h, respectively, in the U‐251 MG cell line (Fig. 3C) and ~20% in the U‐118 GBM cell line at both time points (Fig. 3D). Simultaneously, circARID1A silencing did not affect the linear form of *ARID1A* gene (Fig. 3C,D). The silencing efficiency of circPLOD2 was ~70% after 24 and ~40% after 48 h in the U‐118 MG cell line and did not affect its linear counterpart (Fig. S9A). Even more effective silencing (~80%) both after 24 and 48 h was observed in U‐251 MG cell line, however, in this case we noticed an increased level of mRNA of *PLOD2* gene (Fig. S9B).

In the next step, we preliminary evaluated the biological function of circARID1A and circPLOD2 in GBM cells. Thus, we performed the proliferation assay with U‐118 MG and U‐251 MG cell lines. The results showed a ~43% and ~38.5% decrease in the cell index after circARID1A knockdown in U‐251 MG and U‐118, respectively (Fig. 3E,F). We did not observe any functional relevant changes in proliferation rates after circPLOD2 knockdown (Fig. S9C,D).

The obtained results confirmed the potential biological meaning of circARID1A. Although further detailed analyses are needed to fully characterize those molecules, the performed approach confirms the accuracy of the identification process.

### 3.4 Interplay between circRNAs and RBPs

It is already known that RBPs regulate circRNAs biogenesis via binding to flanking introns and thus promote the back‐splicing process. They can also be sequestered by circRNAs acting as molecular sponges making them direct interactors in molecular processes. This interplay could be significant for the function of both circRNAs and RBPs, therefore we aimed to comprehensively investigate the interplay between those molecules [17, 46, 47, 48, 49].

#### 3.4.1 RBPs might be sequestered by circRNAs

**Fig. 4:**
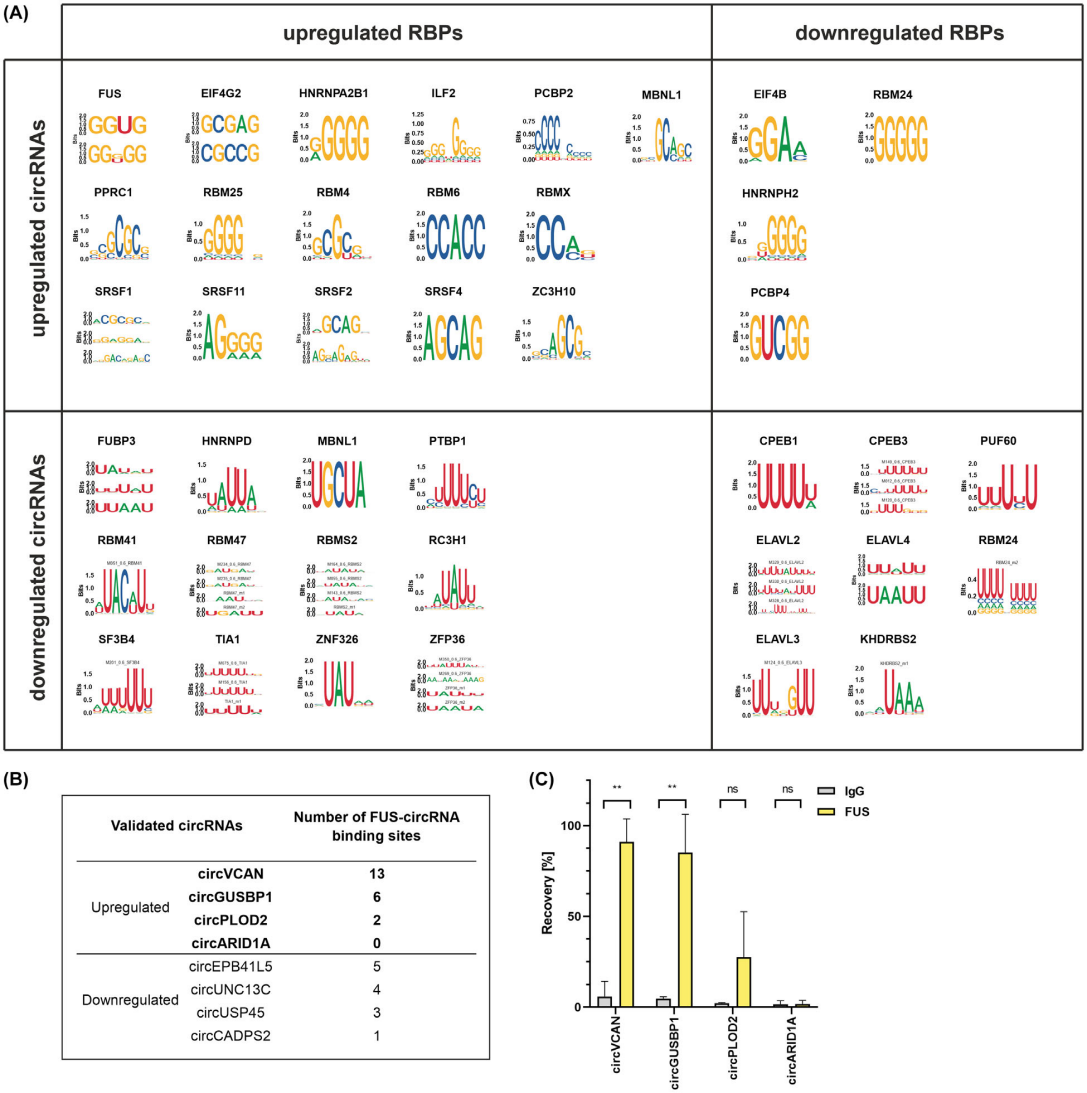
CircVCAN and circGUSBP1 bind to FUS protein. (A) Differentially expressed RNA‐binding proteins (RBPs) with motifs enriched in differentially expressed circular (circRNAs). (B) Number of FUS‐circRNA binding sites in selected upregulated and downregulated circRNAs. (C) RT‐qPCR results of selected circRNAs with putative binding sites in FUS after CLIP. Results are presented as mean values ± SD of 2 biologically independent replicates and normalized to reference HPRT gene. Statistical significance was calculated using one‐way ANOVA test: ns, not statistically significant; * for *P* < 0.05; ** for *P* < 0.01; *** for *P* < 0.001; **** for *P* < 0.0001.

In the first step, we verified the potential probability of circRNAs to sequester RBPs. To examine which differentially expressed RBPs can bind to deregulated circRNAs, we calculated k‐mers (*k* = 6) enrichment in differentially expressed circRNAs in GBM‐PRM vs HB (both up‐ and downregulated) and compared them to each binding motif's position in RBPs. 6‐mers enriched in differentially expressed circRNAs were mapped to the RBPs binding motifs determined either by eCLIP or by RNA‐bind‐n‐seq data [38, 50]. As a result, we have obtained the list of 38 RBPs, predicted to bind deregulated circRNAs, differentially expressed in GBM‐PRM compared to HB tissue. The highly deregulated RBPs comprised, for example, FUS, EIF4G2, or HNRNPA2B1 (Fig. 4A; Fig. S10). We observed that motifs enriched in downregulated circRNAs were mostly U‐rich, whereas those for upregulated ones contained a lot of G and C nucleotides (Fig. 4A) This observation indicated a distinct difference in the recognition sites of upregulated and downregulated circRNAs which might explain the discrimination mechanism between the binding of both types of molecules.

Subsequently, we performed *in silico* search of binding sites for these 38 RBPs in previously validated circRNAs (Table S7). Based on this analysis, we selected FUS protein for further validation. About 13 putative FUS binding sites were found in circVCAN, 6 in circGUSBP1, and 2 in circPLOD2 (Fig. 4B; Table S7). To confirm binding of these 3 circRNAs by FUS, we performed co‐immunoprecipitation using specific anti‐FUS antibody (Fig. S11). We observed enrichment of circVCAN (recovery = 90.98%), circGUSBP1 (recovery = 85.09%), and circPLOD2 (recovery = 27.47%) which confirmed *in silico* prediction (Fig. 4C).

#### 3.4.2 RBPs are correlated with circRNAs and their host gene expression

**Fig. 5:**
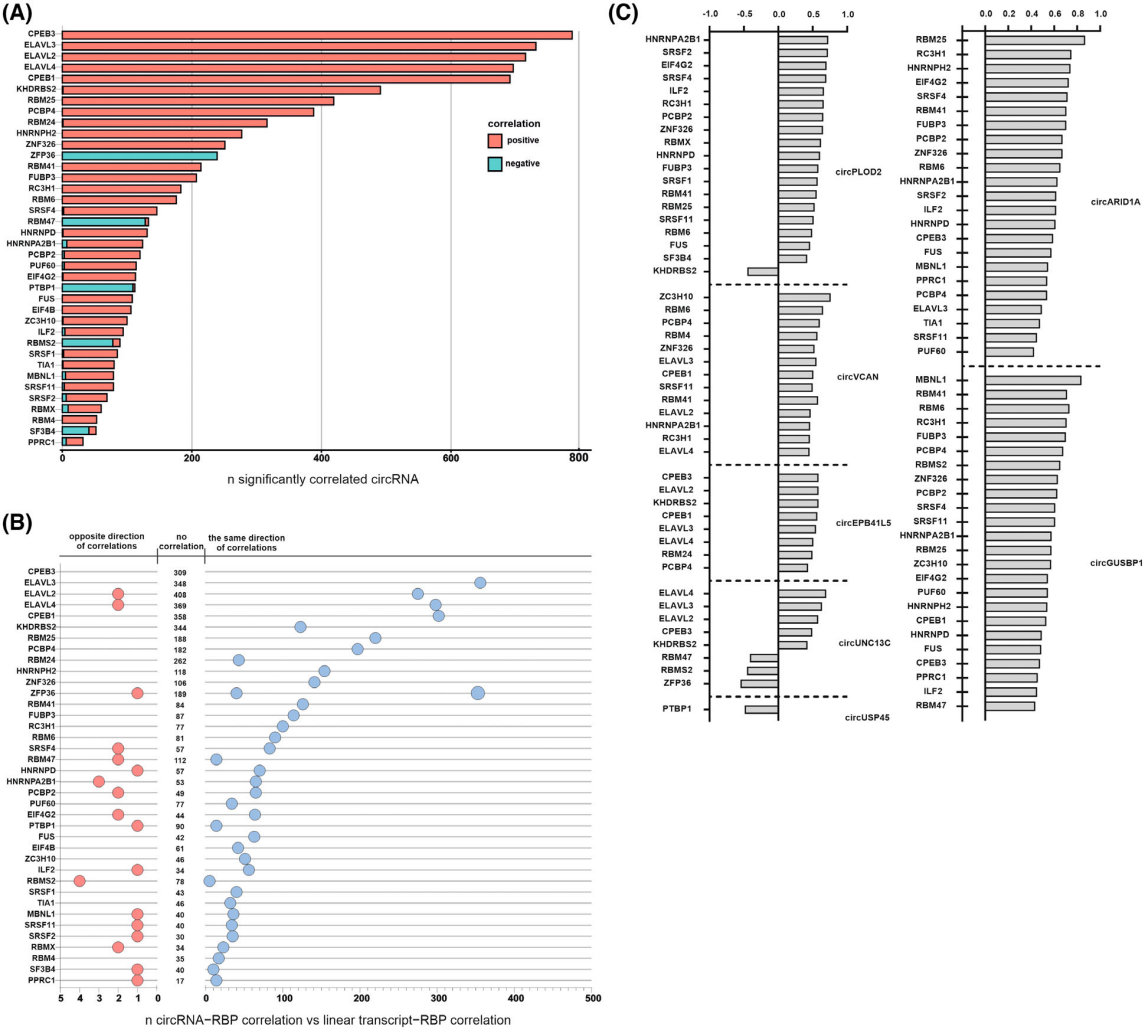
Expression of RBPs is correlated with circRNA and circRNA host genes expression. (A) Bar plot showing the number of circular RNAs (circRNA) significantly correlated with differentially expressed RNA‐binding proteins (RBPs). (B) Dot plot presenting the numbers of circRNAs and the corresponding mRNAs showing opposite correlation direction (left panel, red dots), no correlation (numbers in the middle), or the same correlation direction (right panel, blue dots) with the expression of RBPs from panel A. (C) Correlation of the expression level of selected validated circRNAs with the expression level of dysregulated RBPs. Correlations were assessed using Spearman rank correlation, and statistical significance was calculated using a *t*‐test.

Next, we calculated correlation coefficients between the expression of previously identified differentially expressed RBPs and deregulated circRNA. We observed that expression levels of some RBPs significantly correlate with the expression levels of several hundred differentially expressed circRNAs (Fig. S12). The vast majority exhibited a positive correlation, notably, the ELAV and CPEB families, which were downregulated in GBM (Fig. 5A, marked as red bars). Conversely, ZFP36, RBM47, PTBP1, RBM5S, and SF3B4 mostly showed negative correlation (Fig. 5A, marked as blue bars). Then, we assessed whether the expression of these RBPs correlates with the expression of RNAs' linear counterparts. For this purpose, we used the subset of circRNAs parental mRNAs and calculated coefficients. Furthermore, we confirmed whether the correlation with RBPs has the same or opposite direction considering simultaneously circRNAs and mRNAs. Most of them showed the same direction of correlation in both circRNAs and corresponding mRNAs (Fig. 5B). Interestingly, RBPs positively correlated with circRNA expression (Fig. 5A, marked as red bars) also showed positive correlation with mRNA expression in ~50% of cases. In contrast, RBPs with expression negatively correlated with circRNAs (ZFP36, PTBP1, RBM47, RBMS2, and SF3B4) mostly did not show a significant correlation with mRNA expression. Moreover, we investigated which of the deregulated RBPs might be associated with circRNAs subjected to validation. We observed that upregulated circARID1A and circGUSP1 expression were positively correlated with over 20 RBPs (Fig. 5C).

#### 3.4.3 RBPs might bind with flanking regions thus impact circRNAs biogenesis

**Fig. 6:**
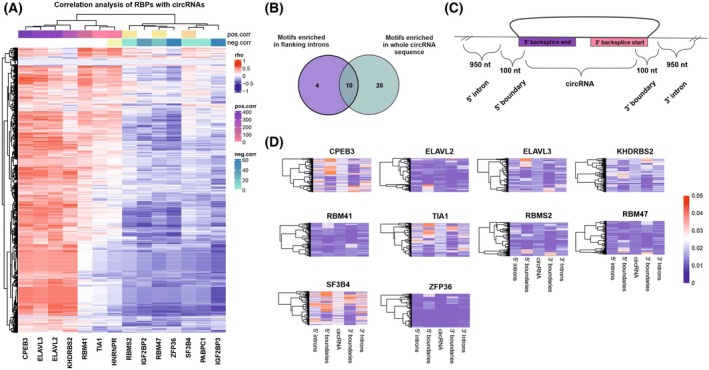
RBPs with motifs enriched in introns flanking circRNAs are correlated with circRNA expression. (A) Heatmap of correlation (rho) between 14 RNA‐binding proteins (RBPs) with enriched motifs flanking backsplice junction of circular RNAs (circRNAs) differentially expressed in glioblastoma (GBM) vs healthy brain (HB). Only significant correlations (*P*‐values <0.05) were reported. For each RBP, number of positively and negatively correlated cicrRNAs were added in the upper bound of the heatmap. (B) Venn diagram showing overlap between RBPs with motifs enriched in flanking introns and in total sequence of circRNA. (C) Schematic representation of circRNA with 5′ and 3′ flanking introns (1000 nt upstream from the 5′ backsplice junction end, 1000 nt downstream from the 3′ backsplice junction end) and 5′ and 3′ boundaries (50 nt from intron and 50 nt from circRNA sequence). (D) Normalized frequency of enriched motifs in RBPs within regions shown in panel C.

As mentioned before, RBPs are important players in circRNA biogenesis [17, 48, 49]. To identify the RBPs potentially involved in circRNAs biogenesis, we analyzed RBP‐binding motifs enriched in flanking introns (up to 1000 nt upstream and downstream from the backsplice junction) of differentially expressed circRNAs in GBM vs HB. We identified 14 differentially expressed RBPs with binding motifs in the flanking introns across all the analyzed samples. CPEB3, ELAVL2, ELAVL3, and KHDRBS2 showed a strong positive correlation with both up‐ and downregulated circRNAs, ELAVL3 was correlated only with downregulated circRNAs. We also noted RBPs with a strong negative correlation with the expression level of upregulated circRNAs like IGF2BP2 and IGF2BP3 (Fig. 6A). In search of potential circularization enhancers, we identified lowly expressed RBPs, which showed a positive correlation with downregulated circRNAs (Fig. S13A,B). CPEB3 was highly correlated with circRLF (*r*‐value = >0.8), circLRBA (*r*‐value = 0.7) and selected before as one of the most downregulated circRNAs in GBM – circEPB41L5 (Fig. S13A), while KHDRBS2 showed strong positive correlation with circLRBA and circVMP1 (Fig. S13B). These findings might indicate RBPs exclusively involved in the regulation of circRNAs biogenesis.

#### 3.4.4 RBPs could both impact circRNAs expression and be sequestered by them

We further investigated whether differentially expressed RBPs could both influence circRNAs biogenesis and be sequestered by the same circRNAs. We found a common set of 10 RBPs: CPEB3, ELAVL2, ELAVL3, KHDRBS2, RBM41, RBM47, RBMS2, SF3B4, TIA1, ZFP36 with motifs enriched in both—flanking introns and total sequence of circRNAs (Fig. 6B; Fig. S14). Additionally, we calculated motif frequencies for these RBPs in the 100 nt regions encompassing the junction site (5′ boundary and 3′ boundary) and we defined intron‐exon and exon‐intron boundaries (Fig. 6C). For most RBPs, we observe similar motif frequencies in introns, boundaries, and circRNA. However, an increased number of binding sites was observed in the sequence covering the 5′ boundary region for the CPEB3 protein. A similar dependence was noted in the case of the SF3B4, which was additionally characterized by an increased frequency of motifs in the 3′ boundary of the circRNA (Fig. 6D). Altogether, it suggests that RBPs might play a dual role—both influence circRNAs biogenesis and at the same time be regulated by these molecules.

### 3.5 Combinatorial analysis of RBPs and circRNAs expression profiles suggest new patient stratification criteria

#### 3.5.1 CircRNA expression is related to the GBM subtypes

**Fig. 7:**
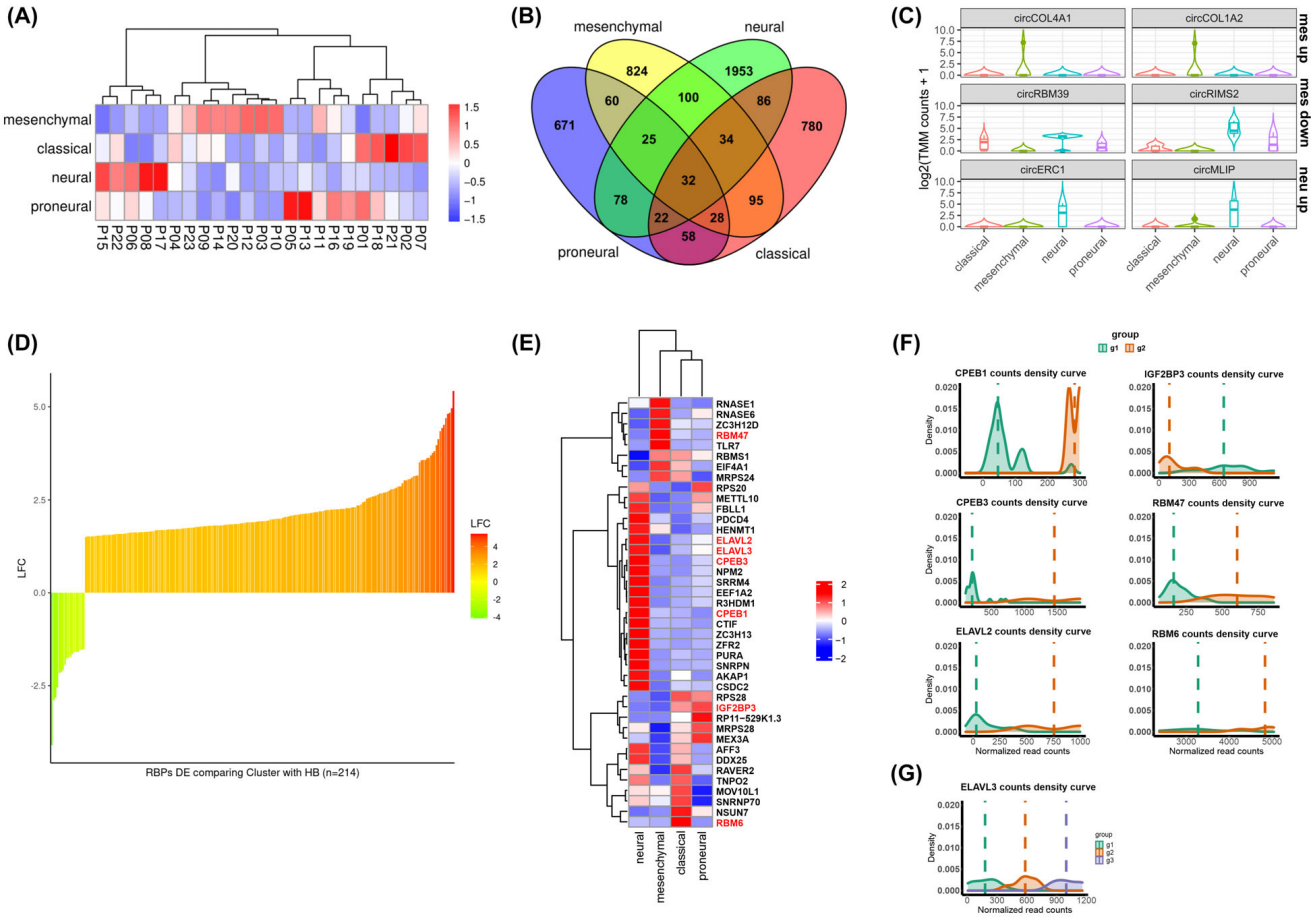
Representation of subtype‐specific circRNA and RBPs clustering GBM into new subtypes. (A) Heatmap presenting subtype classification of the glioblastoma (GBM) samples (*n* = 26) used in the study. (B) Venn diagram showing the number of the identified circular RNAs (circRNAs) in detected GBM subtypes. (C) Comparison of selected circRNA up‐ and downregulated in mesenchymal and upregulated in neural subtypes. (D) Log_2_ fold changes of 214 differentially expressed RNA‐binding proteins (RBPs) that can be used for patients' stratification, comparing GBM samples (*n* = 26) with HB (*n* = 52). (E) Heatmap showing mean expression differences between 41 RBPs within samples belonging to distinct GBM subtypes. RBPs highlighted red contain binding motifs enriched in differentially expressed circRNA sequences/flanking regions. (F, G) RBPs with motifs enriched in differentially expressed circRNA sequences/flanking regions clustering GBM samples into 2 (F) or 3 (G) groups.

We utilized the list of 840 genes from the TCGA GBM dataset and literature [6] as it provides the knowledge for unifying GBM transcriptomic and genomic data allowing for GBM molecular stratification into 4 molecular subtypes: classical, mesenchymal, preneural, and neural. The provided framework allowed us to cluster the analyzed 23 GBM samples into classical (5 samples), mesenchymal (8), neural (5), and proneural (5) (Fig. 7A). Then, we determined the presence of different circRNAs in each of the individual molecular subtypes (Fig. 7B). We identified 54 differentially expressed circRNAs in the neural subtype vs other subtypes, where circERC1 and circMLIP were characterized by the highest expression levels (Fig. 7C; Table S8). The data show the high similarity of the neural subtype to the HB samples. Interestingly, we further distinguished overexpressed circCOL4A1 and circCOL1A2 which were upregulated in the mesenchymal subtype (Table S9). Moreover, in this subtype, we observed decreased expression of circRBM39 and circRIMS2. Noteworthy, we did not find any circRNAs related exclusively to the classical and proneural subtypes.

#### 3.5.2 RBPs in GBM can be used as the new potential stratification markers

To assess also the clinical value of RBPs in GBM, we aimed to identify RBP‐based signatures for patient stratification. At first, we subset RBPs able to discretize patients into two clusters according to their expression with no significant association with GBM molecular subtypes. We identified RBPs that cluster GBM samples into two or more groups (Table S10). Next, we focused on RBPs whose expression in at least one of these newly defined clusters was differentially expressed in comparison to HB samples. We observed a prevalent upregulation of RBPs in the novel subgroups of patients (Fig. 7D). The overall expression of these RBPs was able to separate GBMs from HB samples, with the clusterization of patients in two major newly identified subgroups, independent from the GBM molecular subtypes. We were also looking for RBPs potentially related to the previously mentioned known 4 molecular subtypes and established that there are 41 of them (Fig. S6). From these RBPs, we found 7 with motifs enriched in differentially expressed circRNA and/or flanking sequences (CPEB1, CPEB3, ELAVL2, ELAVL3, IGF2BP3, RBM47, and RBM6) (Fig. 7E, highlighted in red). RBM47 was highly expressed in mesenchymal, RBM6 in classical, and IGF2BP3 in classical and proneural subtypes (Fig. 7E). Majority (6) of 7 selected RBPs further cluster GBM samples into 2 newly defined groups (Fig. 7F) and ELAVL3 cluster GBM samples into 3 groups (Fig. 7G). Thus, RBPs expression profile might be taken into consideration as an additional factor stratifying individual GBM tumors, which could enrich the current division.

#### 3.5.3 RBPs expression correlates with the overall survival rates of GBM patients

Finally, we checked the differentially expressed RBPs and their correlation with the overall survival rates (OVS) of GBM patients. We utilized available data from the TCGA GBM dataset. We selected patients with high or low expression of the given RBPs and found that 30 RBPs differentially expressed in our 23 GBM samples were significantly correlated with OVS in the TCGA GBM cohort (Fig. S15A—upregulated RBPs, S15B—downregulated ones). About 22 of them were previously shown to be brain tumor markers, correlated with disease outcome or response to the treatment (Table S11).

## 4 Discussion

Given that GBM is an aggressive, therapy‐resistant brain tumor with high inter‐ and intratumoral diversity, there is an urgent need to understand the transcriptome profile of this tumor type to find new, distinctive molecular biomarkers and therapeutic targets. Due to their high stability and brain‐specific expression pattern, circRNAs are considered remarkably interesting as potential cancer molecular signatures [51, 52, 53]. Additionally, their role in cancer‐related processes namely proliferation, migration, invasion, and apoptosis in GBM have already been studied and verified [54]. Most of the studies concerning circRNA in GBM are focused on a single or a few molecules and their specific mechanism of action with only a few studies attended to high‐throughput circRNA profiling [55, 56, 57, 58]. Up to now, one study reported the GBM patient‐derived data, however, this study neither solely focus on GBM nor validates the obtained results. The findings reveal that certain circRNAs are deregulated across multiple types of cancers, but did not provide any description of the complex network of interactions and dependencies between circRNAs, their linear counterparts, or RBPs in GBM [59].

In our study, we conducted an extensive RNA‐seq screen focusing on circRNA and RBP expression profiles as well as their interactions. We detected circRNAs differentially expressed in GBM primary tumors (GBM‐PRM) and identified the circRNA progression markers in GBM recurrent ones (GBM‐REC), and additionally, we established the expression profile of RBP genes. Further preliminary functional studies have revealed that circARDI1A—overexpressed in GBM plays role in promoting the proliferation of GBM cells. Subsequent analysis enabled us to generate a comprehensive catalog of circRNA‐RBP interactions including the sequestration of RBPs by circRNA and their involvement in circRNA biogenesis and confirmed direct interactions of FUS protein with circGUSBP1 and circVCAN. Additionally, we demonstrated the clinical potential of circRNAs and RBPs in GBM and identified them as the stratification markers in the *de novo* assembled tumor subtypes. We have identified 1270 circRNAs differentially expressed in GBM‐PRM samples compared to HB controls with almost 90% being downregulated. This pattern aligns with previously published circRNA profiles in different cancer types, including GBM, indicating a potential competition in biogenesis between circRNAs and mRNAs utilized for the protein synthesis—essential for proliferating tumor cells [58, 60, 61, 62, 63]. Further, common downregulation of circRNAs observed in cancer cells can be caused by their extensive proliferation which could dilute the concentration of stable circRNAs [60, 64]. It is also supported by the opposite phenomena, namely the accumulation of circRNA in the nonproliferating aging mouse brain [65] *D. melanogaster* and *C. elegans* aging models [66, 67]. T Tumor cells exhibit high transcription rates, particularly in aggressive cancers like GBM. However, increased back‐splicing occurs when cotranscriptional processing is inhibited or slowed [68, 69]. This aligns with our findings, where circRNA downregulation is largely independent of changes in their linear counterparts, suggesting intact transcription but fewer back‐splicing events.

Previous studies have shown that dysregulation of circRNAs might exert oncogenic functions in GBM, both downregulated in high‐grade glioma circBRAF and upregulated in GBM circPITX1 are associated with poor patients' prognosis [58, 70]. In our study, among downregulated circRNAs, circEBP41L5 was detected as the most decreased one and displays the biggest discrepancy between the expression of linear and circular transcripts. In the literature, circEBP41L5 is described as a GBM suppressor, thus it could serve as a prognostic or therapeutic molecule for new clinical approaches [71]. Remaining downregulated circRNAs might act as suppressors and require further studies to better understand the mechanism underlying GBM.

In the remaining 10% of upregulated circRNA in GBM, the most upregulated was circVCAN which high expression is observed also in gastric cancer [72] and radioresistant glioma tissues [73]. The knockdown of circVCAN resulted in the inhibition of cell proliferation, migration, and invasion, and accelerated apoptosis [72, 73]. Another overexpressed circRNA in our GBM tissues—circPLOD2 was also found to be upregulated in GBM in a previous study [74, 75, 76]. It is also frequently upregulated under hypoxia conditions in HeLa and MCF‐7 cancer cell lines [77]. Subsequent overexpressed circRNA in our study was circARID1A. This molecule seems to be interesting in terms of GBM progression since its function was already established for neuroblastoma cells. It has already been shown that circARID1A promotes cell growth and survival and its action is mediated by direct interactions with the KHSRP RBP [78]. Our functional analysis revealed, that the downregulation of circARID1A has an impact on the decreased proliferation of GBM cells, which suggests its oncogenic function similar to the neuroblastoma cells.

We also put our efforts to distinguish potential molecular markers of GBM progression based on our transcriptomic data. 3 circRNAs: circEGFR, circHLA‐B, circInter6p22 were differentially expressed between GBM‐PRM and GBM‐REC tissue samples. The most overexpressed candidate, particularly in GBM‐REC tumors, was circEGFR. CircEGFR is derived from the EGFR gene which is a well‐established oncogene in various cancers [79, 80]. In contrast, circEFGR was also found as an inhibitor of the malignant progression of glioma [81]. Since there are different circEGFR isoforms originating from different genomic locations, the isoform defined in the mentioned report (hsa_circ_0080223) shows downregulation in tumor tissues, however circEGFR isoform studied in our research (hsa_circ_0080229) is upregulated. Another study, consistent with our findings, indicates that hsa_circ_0080229 promotes glioma tumorigenesis and invasion [82]. This proposes that the expression of circEGFR is increasing with tumor progression. Our results, although observed in a limited group of samples and should be interpreted with caution, supported this hypothesis.

Previous studies revealed that the expression pattern of circRNAs and their circularization are significantly affected by RBPs which bind to the specific motifs in pre‐mRNA [17, 49]. We observed significant changes in the expression of ~half of annotated human RBPome in GBM, which suggests that some of the changes in circRNA expression may be caused by disruption of their biogenesis. Moreover, these direct interactions of circRNAs with RBPs can substantially affect molecular processes [49]. Additionally, circRNAs can act as protein sponges, decoys that regulate protein accessibility in cell compartments, and also as scaffolds that allow protein–protein interactions mediating both circRNA and RBP functions [48]. Based on these findings, we analyzed the transcriptome of RBPs, identifying enriched recognition motifs for 38 RBPs in deregulated circRNAs. In our study, upregulated circRNAs were mostly motifs enriched in G and C in differentially expressed RBPs. It was shown that RBPs enriched in G‐rich motifs might be splicing activators, that might potentially explain the upregulation of circRNAs to which they bind [26]. It has been already shown that FUS binds to introns to flank the back‐splicing junctions, which affects circRNA expression in murine embryonic stem cell‐derived motor neurons [83]. Another study showed in the case of FUS protein, the specificity of RBP‐RNA binding becomes much higher for circRNA than for linear RNA. [84] Following, we selected nuclear FUS protein to validate its potential interactions with upregulated circRNAs: circVCAN, circGUSBP1, circPLOD2, and circARID1A. Conducted co‐immunoprecipitation experiment confirmed FUS binding with nuclear circVCAN and circGUSBP1 and did not with circARID1A, which is convergent with circARID1A cytoplasmic localization.

On the other hand, downregulated circRNAs are enriched in U‐rich motifs. It was previously shown that U‐rich motifs correlate with higher mRNA decay rates [85]. Our further analyses showed that expression of RBPs from ELAV family, downregulated in GBM, is correlated with the highest number of downregulated circRNAs. ELAVL proteins recognize AU‐rich elements in the 3’ UTRs and thereby regulate gene expression post‐transcriptionally stabilizing mRNA to avoid degradation [86]. Thus, decreased expression of ELAV proteins can at least partially explain lower expression of their putative targets.

Additionally, we found ELAVL2 and ELAVL3 motifs enrichment in flanking introns with the highest frequency in a region covering 100 nt upstream from the 5′ backsplice junction end. Another protein family that might impact circRNAs expression is CPEB. Both CPEB1 and CPEB3 are downregulated in GBM with U‐rich motifs enriched in downregulated circRNAs and show the highest number of positive correlated downregulated circRNAs including validated by us circEPB41L5.

Apart from the transcriptome‐wide characterization of circRNAs and RBPs in GBM and their putative interactions, we extended our analysis to determine differentially expressed circRNAs and RBPs specific for four known molecular GBM subtypes distinguished according to mutation landscape and gene expression pattern [6].

According to the circRNA expression pattern, the neural subtype was the most similar to the HB samples. The RBPs expression analysis revealed the high expression levels of CPEB and ELAVL families in neural GBM samples which are generally downregulated in GBM [87, 88]. These findings confirm earlier reports that the neural subtype may be nontumor margins contamination [89, 90, 91].

Among GBM subtypes, the mesenchymal one is known as the most aggressive, invasive, and resistant to treatment [92]. We found that both circCOL4A1 and circCOL1A2 are highly expressed in this subtype. Moreover, circCOL1A2 was previously described as upregulated in gastric cancer enhancing the migration and invasion properties [93] and also promoting angiogenesis in diabetic retinopathy [94, 95], which can make circCOL1A2 a relevant marker of mesenchymal GBM subtype. For the first time, we managed to distinguish new GBM groups based on RBP expression patterns, independent of known molecular subtypes. Again, among other RBPs we found ELAVL family stratifies GBM into 2 or 3 groups. Taken together this RBP family, previously shown as involved in neural development may be also very interesting in the context of circRNA biogenesis and/or regulation as well as GBM patients' stratification.

## 5 Conclusions

Specifically, we established a list of circRNAs differentially expressed in GBM‐PRM tumors, as well as the circRNAs progression markers in GBM‐REC samples, and indicated a global deregulation of genes encoding RBPs. Selected circRNAs were further verified experimentally by the qRT‐PCR, and their subcellular localization was also described. Subsequent preliminary functional analysis showed that circARID1A can be involved in proliferation of GBM cells which may suggest its oncogenic function in this cancer. Further analysis allowed us to generate a comprehensive catalog of circRNA‐RBP interactions regarding both the RBPs sequestration by circRNA as well as the RBPs involvement in circRNA biogenesis. Based on the co‐immunoprecipitation approach, we could identify FUS protein direct interaction with circVCAN and circGUSPB1.

Furthermore, we demonstrated the clinical potential of circRNAs and RBPs in GBM and proposed them as the stratification markers in the *de novo* assembled tumor subtypes.

Moreover, our findings suggest that both circRNAs and RBPs might be considered as clinical markers and tumor‐subtyping factors in the future. The list of potential functions of distinct circRNAs as well as the circRNA‐RBP interactions is long and their regulatory functions are complex. The mechanism of action and the mutual dependencies await further in‐depth investigation. However, with our comprehensive study, we provide a strong basis for future research into the molecular mechanisms and clinical implications of circRNAs and RBPs interactions in GBM.

## Conflict of interest

The authors declare no conflict of interest.

## Author contributions

JLŁ, ZZ, MPS, KK. JOM, AG, and KR: conceptualization; RP and AMB: sample collection; JLŁ, ZZ, MPS, JOM, AG, KK, PG, AB, MZ, JK, AR‐W, and KR: formal analysis; JLŁ, ZZ, MPS, KK, AG, AB, PG, and KR: investigation; JLŁ, ZZ, MPS, AB, SB, AR‐W, and MZ: methodology; KR: resources; KR: supervision; KR: funding acquisition; MPS and AB: visualization; JLŁ, ZZ, MPS, and JOM: writing—original draft; JLŁ, ZZ, MPS, JOM, AG, KK, AB, SB, AR‐W, and KR: writing—review and editing. All authors have read and approved the final version of the manuscript.

## Peer review

The peer review history for this article is available at https://www.webofscience.com/api/gateway/wos/peer‐review/10.1002/1878‐0261.70005.

## Supporting information


**Fig. S1.** Electrophoretic separation of total RNA isolated from healthy brain (HB) samples.
**Fig. S2.** Electrophoretic separation of total RNA isolated from primary glioblastoma (GBM‐PRM) and recurrent glioblastoma (GBM‐REC) samples.
**Fig. S3.** Overview of the features of circRNAs identified in primary glioblastoma (GBM‐PRM), recurrent glioblastoma (GBM‐REC) and healthy brain (HB).
**Fig. S4.** Histogram showing percentage of unique circular RNA (circRNA) per chromosome in primary glioblastoma (GBM‐PRM), recurrent glioblastoma (GBM‐REC) and healthy brain (HB).
**Fig. S5.** Clustered heatmap illustrating differential expression of circular RNAs (circRNAs) among glioblastoma (GBM), primary glioblastoma (GBM‐PRM) and recurrent glioblastoma (GBM‐REC) vs healthy brain (HB) samples presented as log_2_(fold change) including samples classification to the molecular GBM subtypes according to Verhaak et al.
**Fig. S6.** Clustered heatmap illustrating differential expression of RNA‐binding proteins (RBPs) among glioblastoma (GBM), primary glioblastoma (GBM‐PRM) and recurrent glioblastoma (GBM‐REC) vs healthy brain (HB) (left panel) and different profiles of RBP expression between GBM samples (right panel) presented as log_2_(fold change).
**Fig. S7.** RNase R treatment of circular RNAs (circRNAs) and their linear counterparts.
**Fig. S8.** (A) Log_2_ fold change comparison of selected circRNAs dysregulated in primary glioblastoma (GBM‐PRM) based on qRT‐PCR and RNA‐seq analysis. (B) Log_2_ fold change comparison of selected circRNAs dysregulated in GBM‐REC based on qRT‐PCR and RNA‐sequencing analysis. (C) Pearson correlation of expression for validated circRNAs and their linear counterparts dysregulated in GBM‐PRM. D. Pearson correlation of expression for validated circRNAs and their linear counterparts dysregulated in GBM‐REC.
**Fig. S9.** Proliferation rates after circPLOD2 knock‐down in glioblastoma (GBM) cells.
**Fig. S10.** Normalized expression comparison of RNA‐binding proteins’ (RBPs’) genes in healthy brain (HB) and glioblastoma (GBM).
**Fig. S11.** Western blot experiment confirmed the presence of FUS protein in immunoprecipitated complexes.
**Fig. S12.** Boxplots representing correlation coefficient between RNA‐binding proteins (RBPs) with motifs enriched in circRNAs and differentially expressed circRNAs.
**Fig. S13.** RNA‐binding proteins (RBPs) with motifs enriched in introns flanking circular RNAs (circRNAs) are correlated with circRNA expression.
**Fig. S14.** Upset plot for RNA‐binding proteins (RBPs) with binding motifs in flanking introns vs RBPs with binding motifs in circular RNAs (circRNAs).
**Fig. S15.** Survival analysis for differentially expressed RNA‐binding proteins (RBPs) based on TCGA glioblastoma (GBM) dataset.

**Table S1.** The characteristics of glioblastoma (GBM) tissue donors, who donated their tissue to the study. Patient ID: P – primary GBM, R – recurrent GBM; Classification of Malignant Tumors (TNM) according to Union for International Cancer Control (UICC) – T1, T2, T3, T4 represent size and/or extension of the primary tumor; RNA Integrity Number (RIN); samples subjected to experimental validation marked in blue.

**Table S2.** The characteristics of healthy brain (HB) controls used in the study. RNA Integrity Number (RIN).

**Table S3.** Differentially expressed RNA‐binding proteins (RBPs) used for survival analysis.

**Table S4.** List of siRNA sequences used in knockdown experiments.

**Table S5.** List of the primers used in the qPCR validation and RNase R treatment experiment.

**Table S6.** Characteristics of the circular and linear transcripts subjected to the validation. ns, no statistical significance for *P* ≥ 0.05.

**Table S7.** Number of RNA‐binding protein‐circular RNA (RBP‐circRNA) binding sites in validated circRNAs.

**Table S8.** Circular RNAs (circRNAs) differentially expressed in neural subtype vs other subtypes.

**Table S9.** Circular RNAs (circRNAs) differentially expressed in mesenchymal subtype vs other subtypes.

**Table S10.** Differentially expressed RNA‐binding proteins (RBPs) used for stratification of glioblastoma (GBM) samples into different groups.

**Table S11.** RNA‐binding proteins (RBPs) shown to be brain tumor markers, correlated with disease outcome or response to treatment.

## Data Availability

Sequencing data have been deposited in NCBI GEO repository under accession code GSE196695. Scripts used for data analysis and processed data are available at https://github.com/MSajek/GBM_circs.
