# Supplementary material for: Transcriptome‐wide analysis of circRNA and RBP profiles and their molecular relevance for GBM
Source: Mol Oncol. 2025 Feb 26;19(8):2270–91. doi: 10.1002/1878-0261.70005 (PMC12330939; doi:10.1002/1878-0261.70005)
Supplement: Supplementary file 1 — Fig. S1. Electrophoretic separation of total RNA isolated from healthy brain (HB) samples. Fig. S2. Electrophoretic separation of total RNA isolated from primary glioblastoma (GBM‐PRM) and recurrent glioblastoma (GBM‐REC) samples. Fig. S3. Overview of the features of circRNAs identified in primary glioblastoma (GBM‐PRM), recurrent glioblastoma (GBM‐REC) and healthy brain (HB). Fig. S4. Histogram showing percentage of unique circular RNA (circRNA) per chromosome in primary glioblastoma (GBM‐PRM), recurrent glioblastoma (GBM‐REC) and healthy brain (HB). Fig. S5. Clustered heatmap illustrating differential expression of circular RNAs (circRNAs) among glioblastoma (GBM), primary glioblastoma (GBM‐PRM) and recurrent glioblastoma (GBM‐REC) vs healthy brain (HB) samples presented as log2(fold change) including samples classification to the molecular GBM subtypes according to Verhaak et al. Fig. S6. Clustered heatmap illustrating differential expression of RNA‐binding proteins (RBPs) among glioblastoma (GBM), primary glioblastoma (GBM‐PRM) and recurrent glioblastoma (GBM‐REC) vs healthy brain (HB) (left panel) and different profiles of RBP expression between GBM samples (right panel) presented as log2(fold change). Fig. S7. RNase R treatment of circular RNAs (circRNAs) and their linear counterparts. Fig. S8. (A) Log2 fold change comparison of selected circRNAs dysregulated in primary glioblastoma (GBM‐PRM) based on qRT‐PCR and RNA‐seq analysis. (B) Log2 fold change comparison of selected circRNAs dysregulated in GBM‐REC based on qRT‐PCR and RNA‐sequencing analysis. (C) Pearson correlation of expression for validated circRNAs and their linear counterparts dysregulated in GBM‐PRM. D. Pearson correlation of expression for validated circRNAs and their linear counterparts dysregulated in GBM‐REC. Fig. S9. Proliferation rates after circPLOD2 knock‐down in glioblastoma (GBM) cells. Fig. S10. Normalized expression comparison of RNA‐binding proteins’ (RBPs’) genes in healthy brain ( [file MOL2-19-2270-s012.pdf]

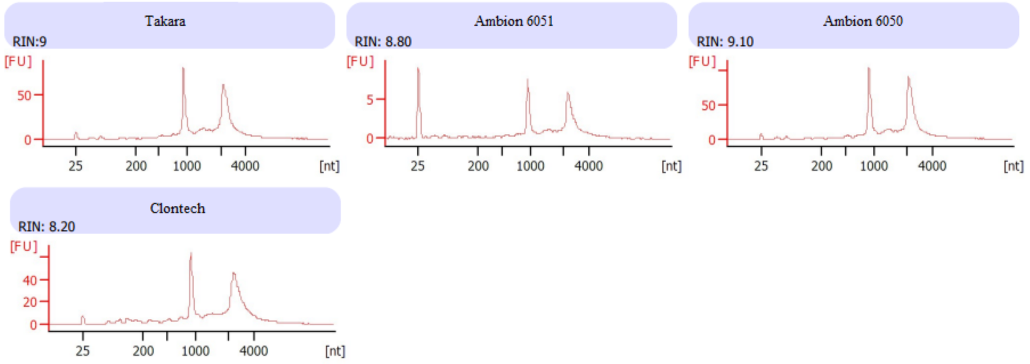

Supplementary Figure 1. Electrophoretic separation of total RNA isolated from healthy brain (HB) samples. The electrophoretic separation was prepared using the Agilent 2100 Bioanalyzer system. Only samples with RIN (RNA Integrity Number) value above 8 were taken into further investigation.

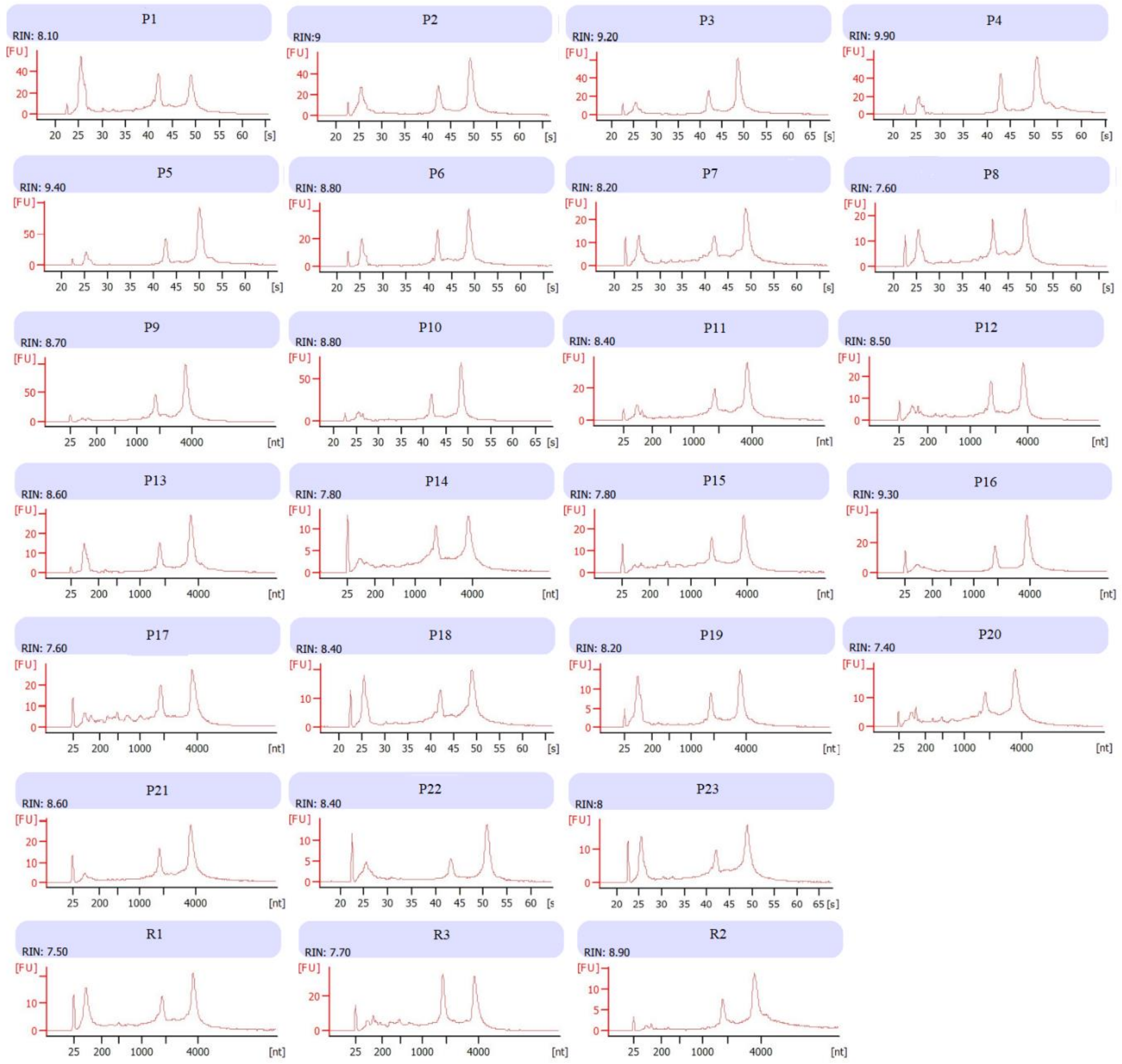

Supplementary Figure 2. Electrophoretic separation of total RNA isolated from primary glioblastoma (GBM-PRM) and recurrent glioblastoma (GBM-REC) samples. Electrophoretic separation was prepared using the Agilent 2100 Bioanalyzer system. Only samples with RIN (RNA Integrity Number) value above 7 were taken into further investigation.

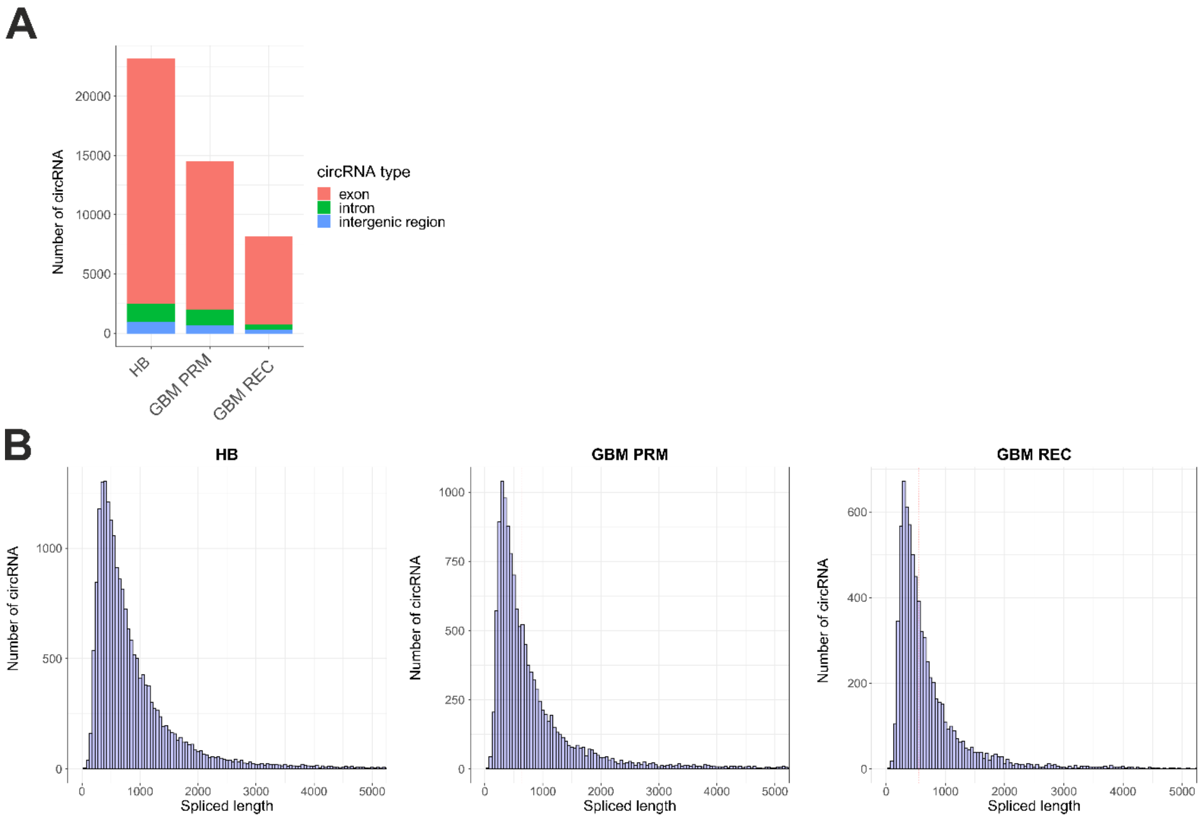

Supplementary Figure 3. Overview of the features of circRNAs identified in primary glioblastoma (GBM-PRM), recurrent glioblastoma (GBM-REC) and healthy brain (HB). A. Stacked barplot presents the genomic origin of identified circular RNAs (circRNAs). Vast majority are derived from exons. **B.** Histograms displaying a spliced length distribution of identified circRNAs for analyzed tissues.

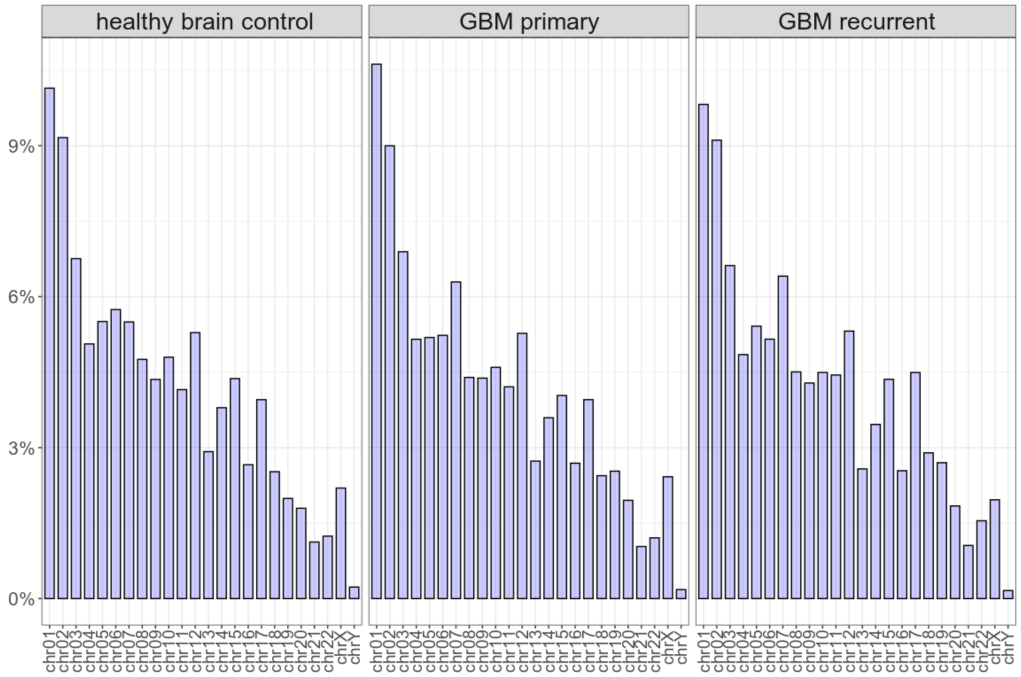

Supplementary Figure 4. Histogram showing percentage of unique circular RNA (circRNA) per chromosome in primary glioblastoma (GBM-PRM), recurrent glioblastoma (GBM-REC) and healthy brain (HB).

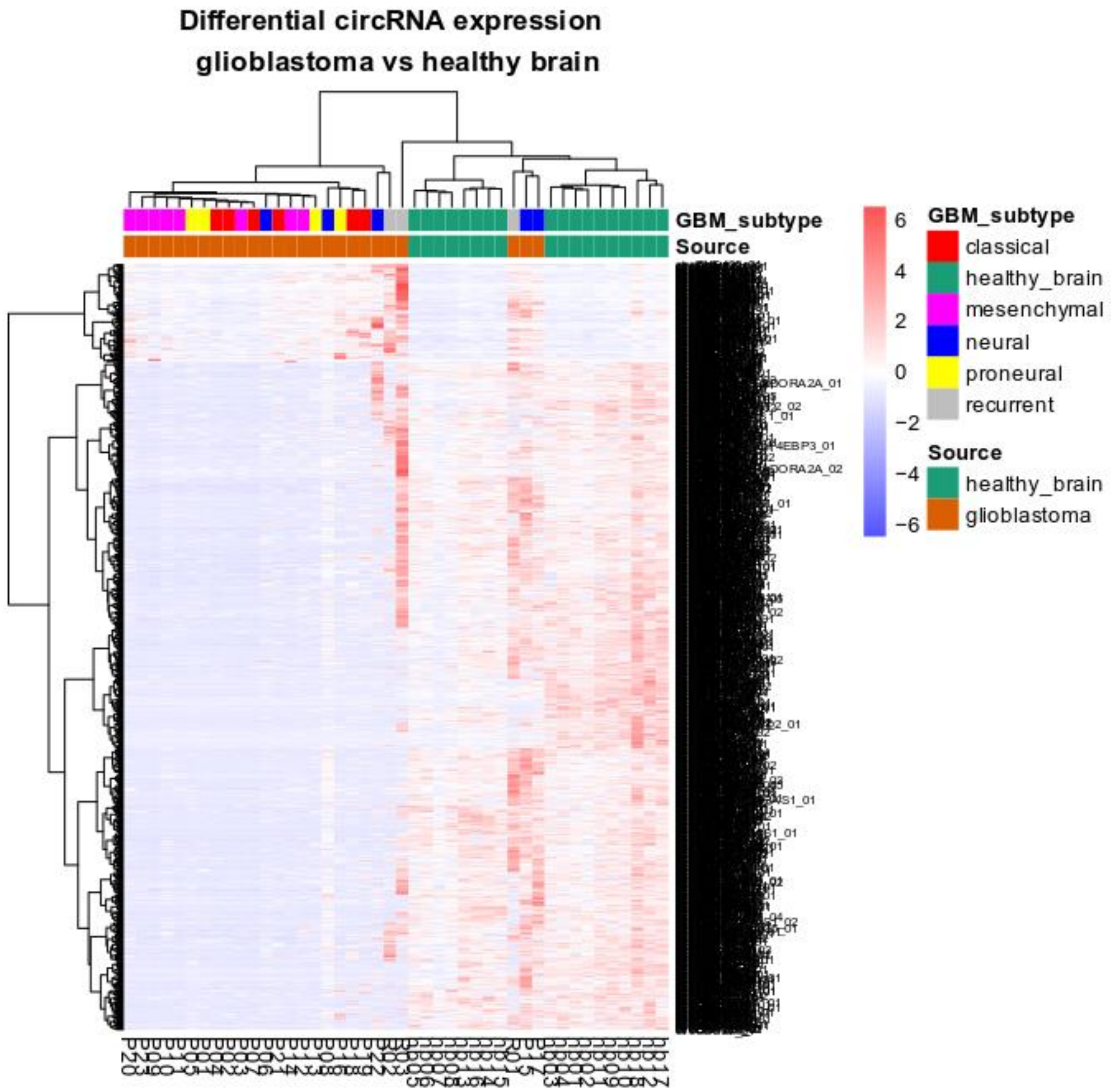

Supplementary Figure 5. Clustered heatmap illustrating differential expression of circular RNAs (circRNAs) among glioblastoma (GBM) (primary glioblastoma (GBM-PRM) and recurrent glioblastoma (GBM-REC) versus healthy brain (HB) samples presented as log2(fold change) including samples classification to the molecular GBM subtypes according to Verhaak et al.

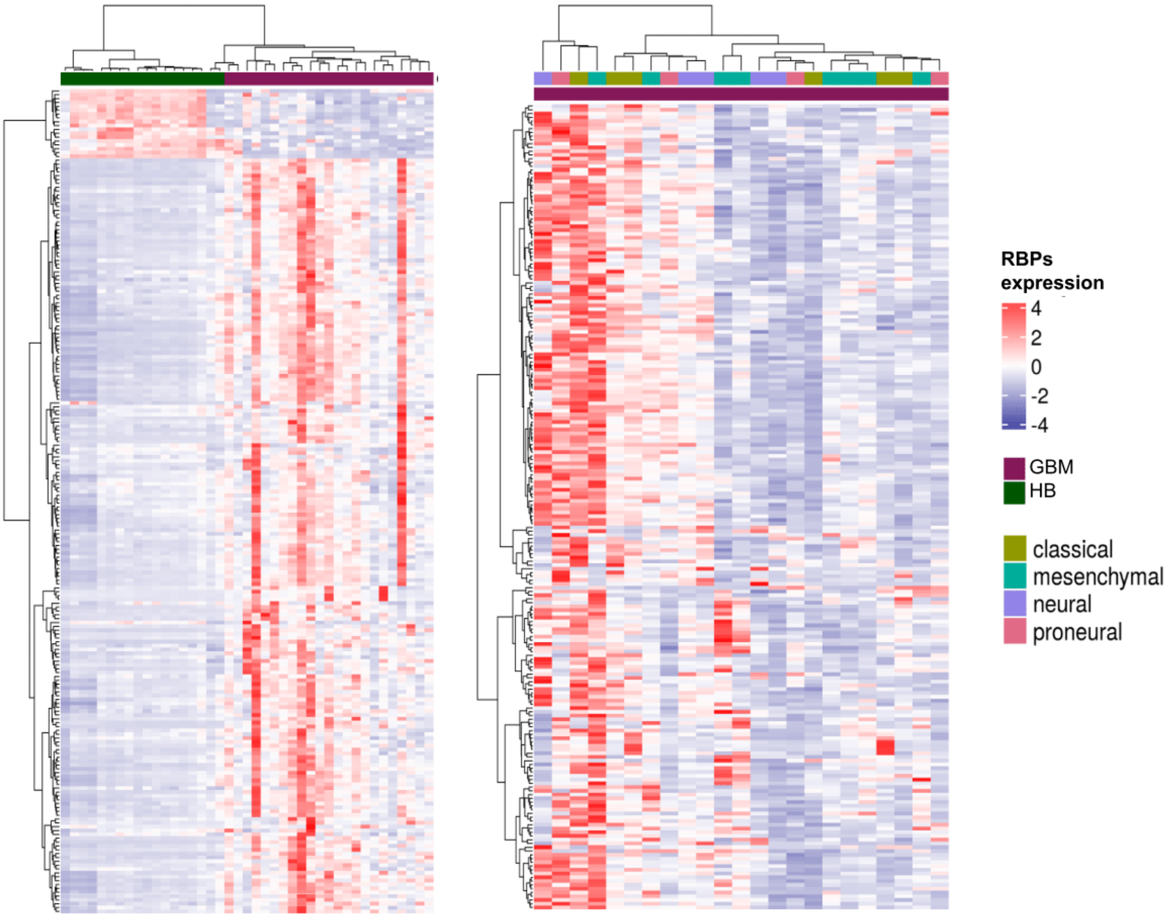

Supplementary Figure 6. Clustered heatmap illustrating differential expression of RNA-binding proteins (RBPs) among glioblastoma (GBM), primary glioblastoma (GBM-PRM) and recurrent glioblastoma (GBM-REC) versus healthy brain (HB) (left panel) and different profiles of RBP expression between GBM samples (right panel) presented as log2(fold change). Right panel represents the subset of left panel. Expression of 214 RBPs can be used to define novel patients' subgroups beyond known molecular subtypes.

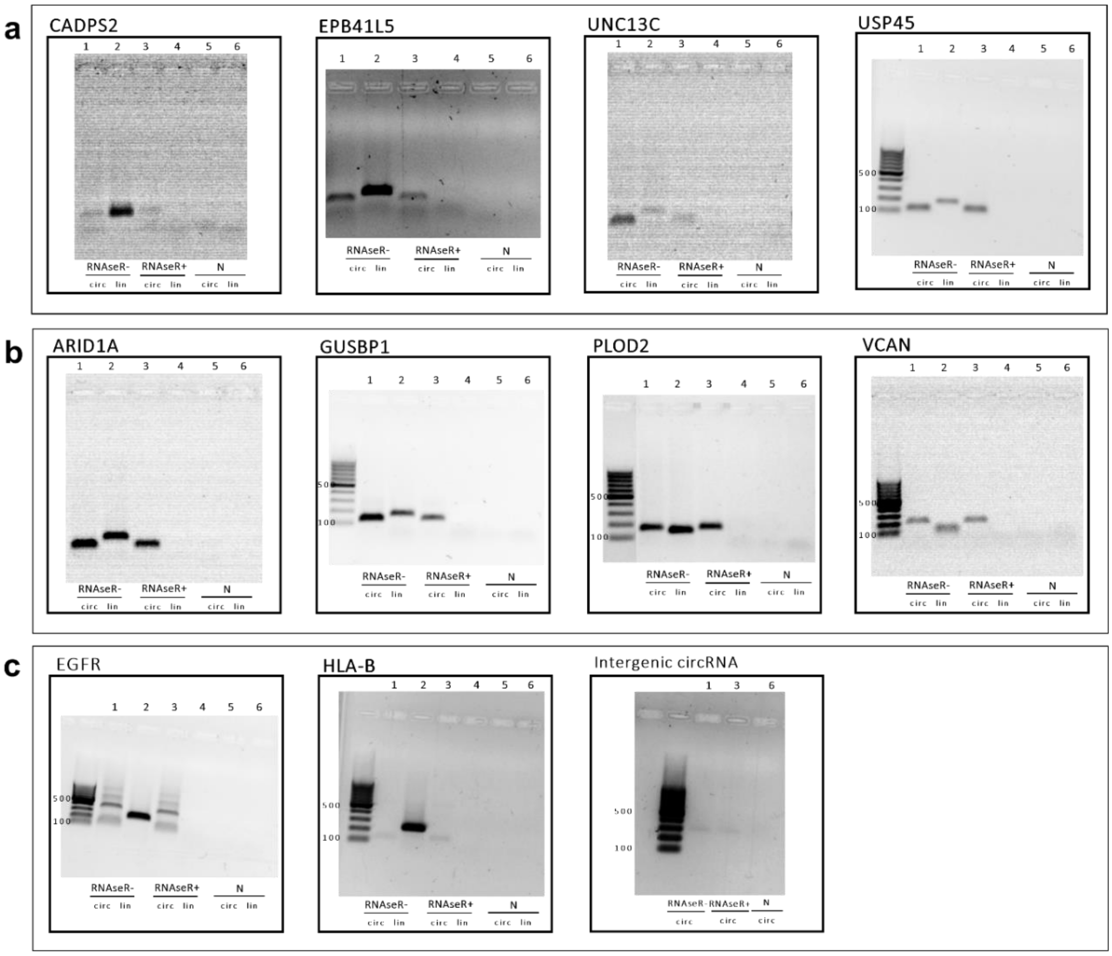

Supplementary Figure 7. RNase R treatment of circular RNAs (circRNAs) and their linear counterparts. A-C. Bands represent PCR products on agarose gel of selected downregulated, upregulated and progression-related candidates, respectively. **1.** Band represent PCR product on agarose gel of circRNA without RNase R treatment; **2.** Band represent PCR product on agarose gel of mRNA without RNase R treatment; **3.** Band represent PCR product on agarose gel of circRNA with RNase R treatment; **4.** Band represent PCR product on agarose gel of mRNA with RNase R treatment; **5, 6.** Negative control of PCR product.

A

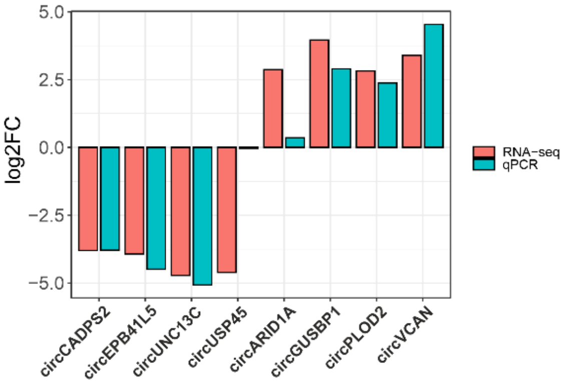

B

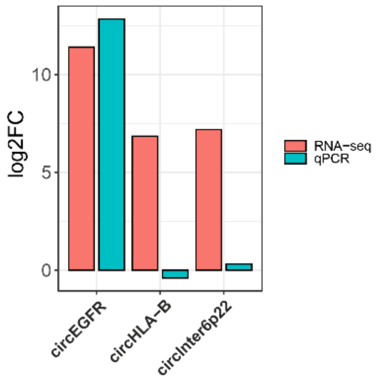

C

Gene ID	Pearson Correlation Coefficient (r)	p-value
CADPS2	0.1440	0.7336
EPB41L5	0.1440	0.5504
UNC13C	0.9970	<0.0001
USP45	-0.5331	0.1737
ARID1A	0.2221	0.2385
GUSBP1	0.4587	0.2529
PLOD2	0.4497	0.2636
VCAN	0.6957	0.05533

D

Gene ID	Pearson Correlation Coefficient (r)	p-value
EGFR	0.9175	0.003587
HLA-B	0.8896	0.007315
circInter6p22	-	-

Supplementary Figure 8. A. Log2 fold change comparison of selected circRNAs dysregulated in primary glioblastoma (GBM-PRM) based on qRT-PCR and RNA-seq analysis. **B.** Log2 fold change comparison of selected circRNAs dysregulated in GBM-REC based on qRT-PCR and RNA-sequencing analysis. **C.** Pearson correlation of expression for validated circRNAs and their linear counterparts dysregulated in GBM-PRM. **D.** Pearson correlation of expression for validated circRNAs and their linear counterparts dysregulated in GBM-REC.

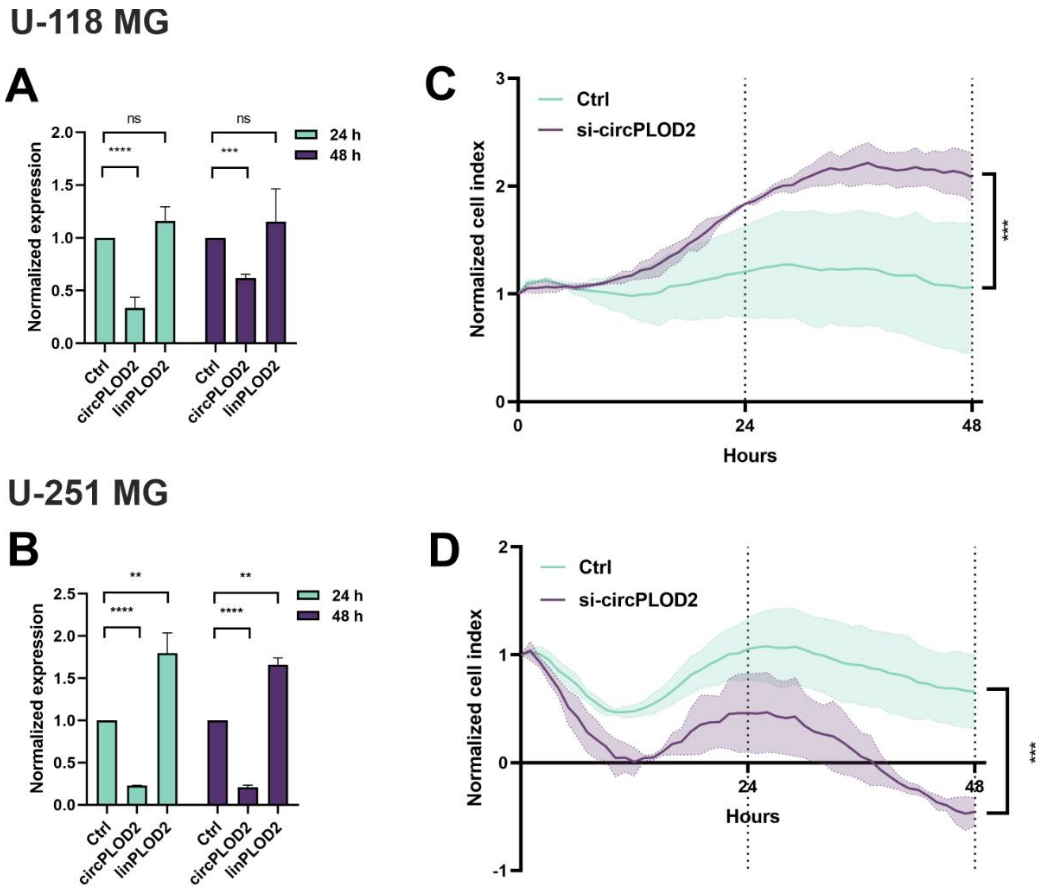

Supplementary Figure 9. Proliferation rates after circPLOD2 knock-down in glioblastoma (GBM) cells. A-B. Normalized expression level of circPLOD2 and linPLOD2 after knock-down of circPLOD2 in U-118 MG (**A**) and U-251 MG (**B**) GBM cell lines after 24 and 48 hours. The efficiency of the downregulation was established by qRT-PCR analysis and scrambled siRNA served as a control (Ctrl). **C-D.** Proliferation rates after circPLOD2 knock-down in U-118 MG (**C**) and U-251 MG (**D**) GBM cell lines. Results are presented as mean values ± SD, normalized to reference *HPRT* gene and compared to control. Statistical significance was calculated using One-Way ANOVA test: ns – not statistically significant; * for $p < 0.05$; ** for $p < 0.01$; *** for $p < 0.001$; **** for $p < 0.0001$.

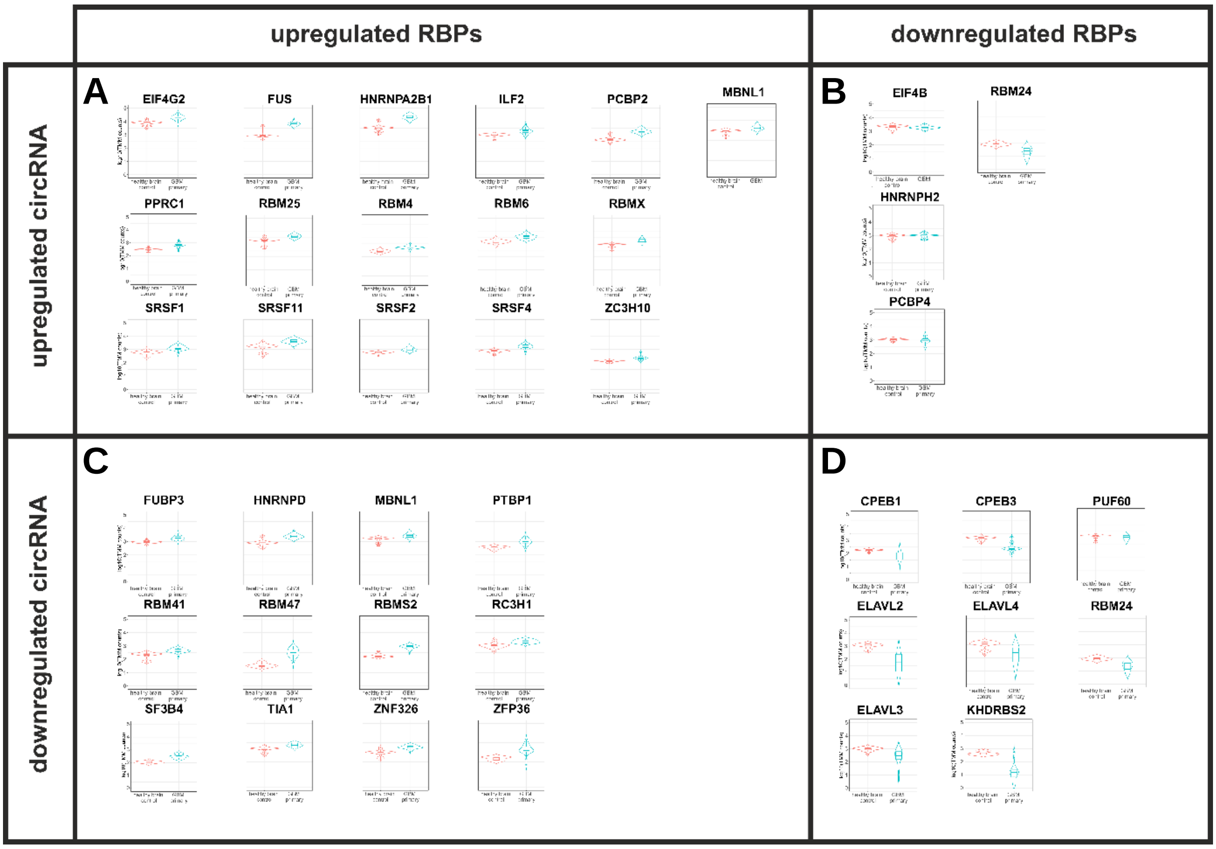

Supplementary Figure 10. Normalized expression comparison of RNA-binding proteins' (RBPs') genes in healthy brain (HB) and glioblastoma (GBM). A. Upregulated RBPs with motifs enriched in upregulated circular RNAs (circRNAs). **B.** Downregulated RBPs with motifs enriched in upregulated circRNAs. **C.** Upregulated RBPs with motifs enriched in downregulated circRNAs. **D.** Downregulated RBPs with motifs enriched in downregulated circRNAs. Sequence logos represent motifs enriched in circRNAs.

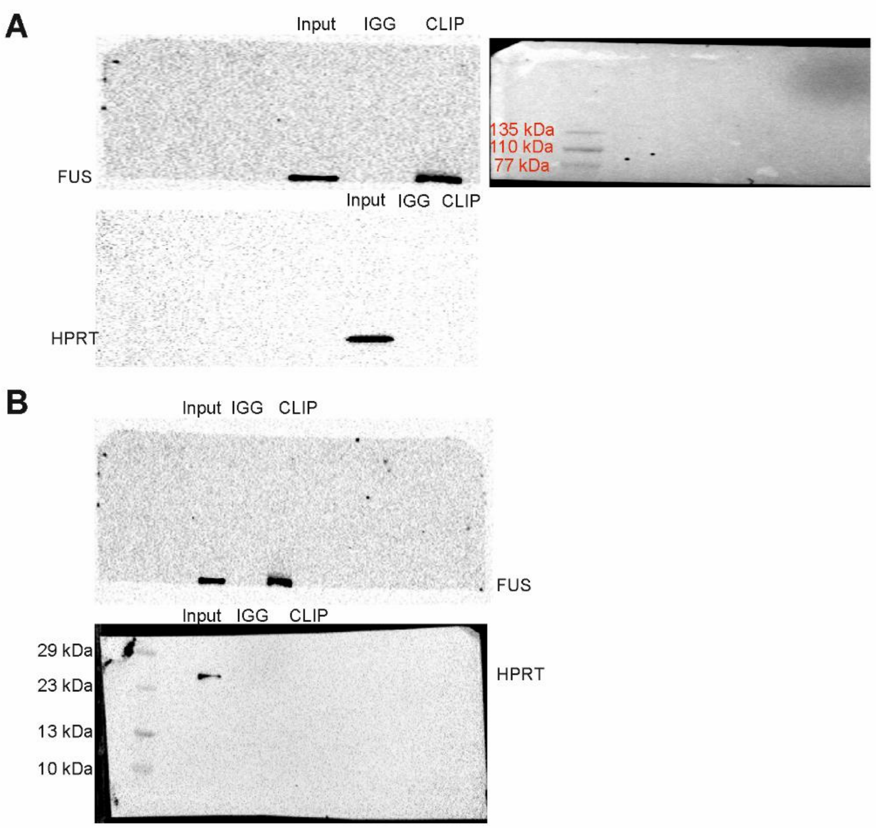

Supplementary Figure 11. Western blot experiment confirmed the presence of FUS protein in immunoprecipitated complexes. A-B. Bands represent FUS and HPRT proteins in an FUS-based cross-linking and immunoprecipitation (CLIP) experiment. FUS was detected in the input, IgG, and CLIP fractions, while HPRT was detected in the input.

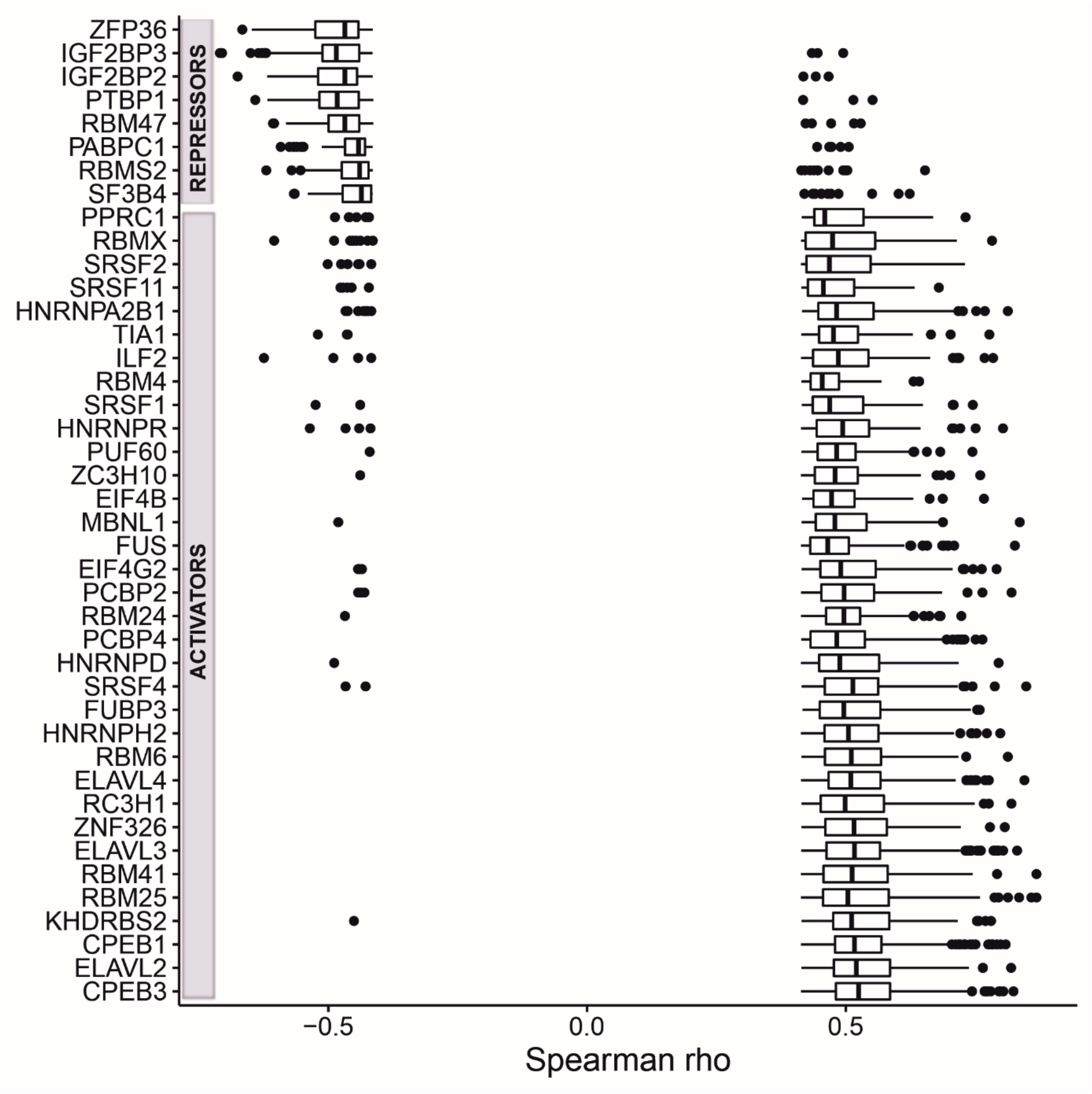

Supplementary Figure 12. Boxplots representing correlation coefficient between RNA-binding proteins (RBPs) with motifs enriched in circRNAs and differentially expressed circRNAs.

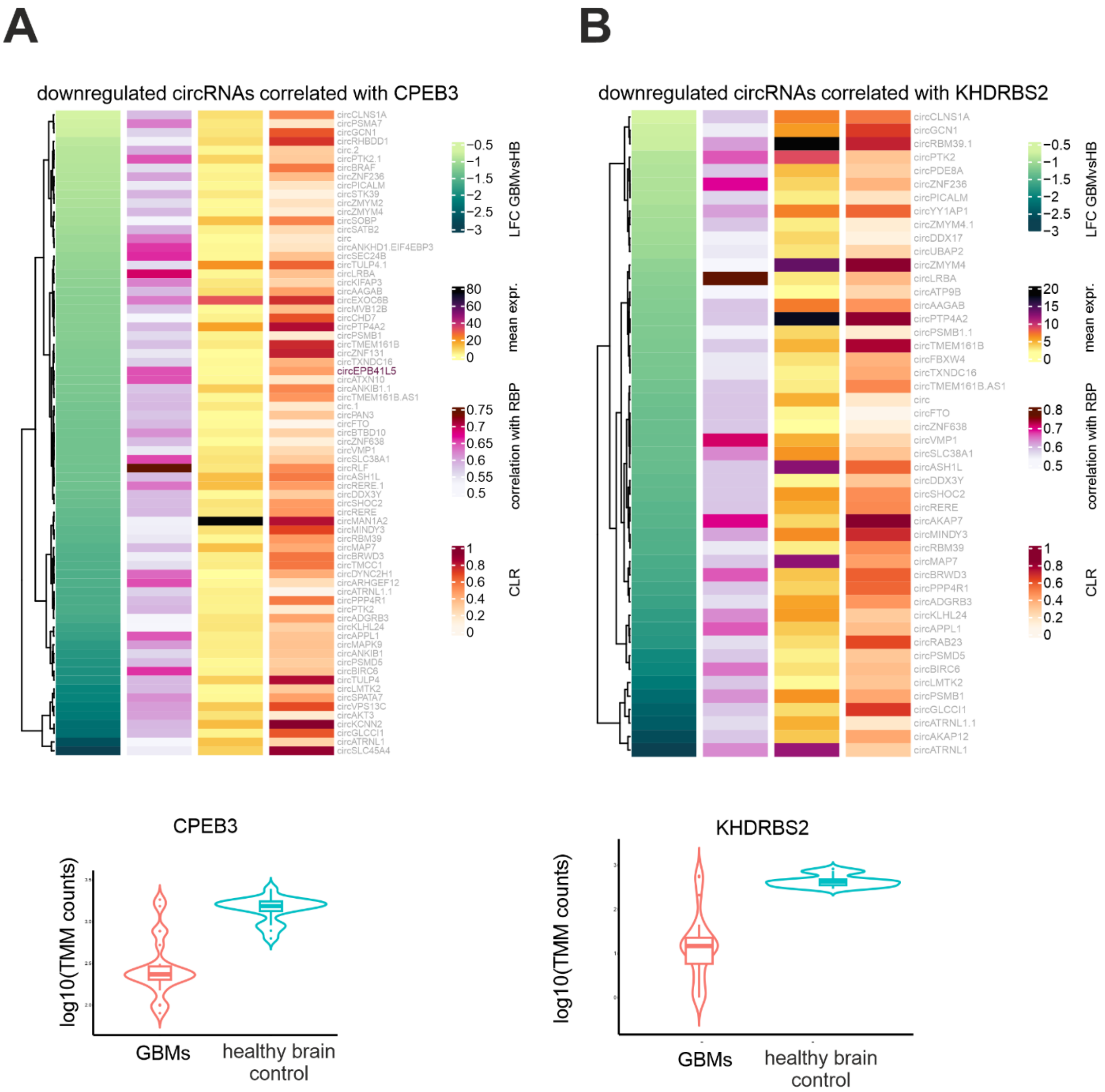

Supplementary Figure 13. RNA-binding proteins (RBPs) with motifs enriched in introns flanking circular RNAs (circRNAs) are correlated with circRNA expression. A-B. CBEP3 and KHDRBS2 normalized expression distribution in healthy brain (HB) and glioblastoma (GBM). For each RBP, correlated circRNAs are reported in terms of: I. average normalized expression in GBM samples; II. average circular-to-linear-ratio (CLR) in GBM; III. log2fold change comparing circRNA expression in GBM vs HB and IV. correlation entity with the RBP - CBEP3 and KHDRBS2, respectively.

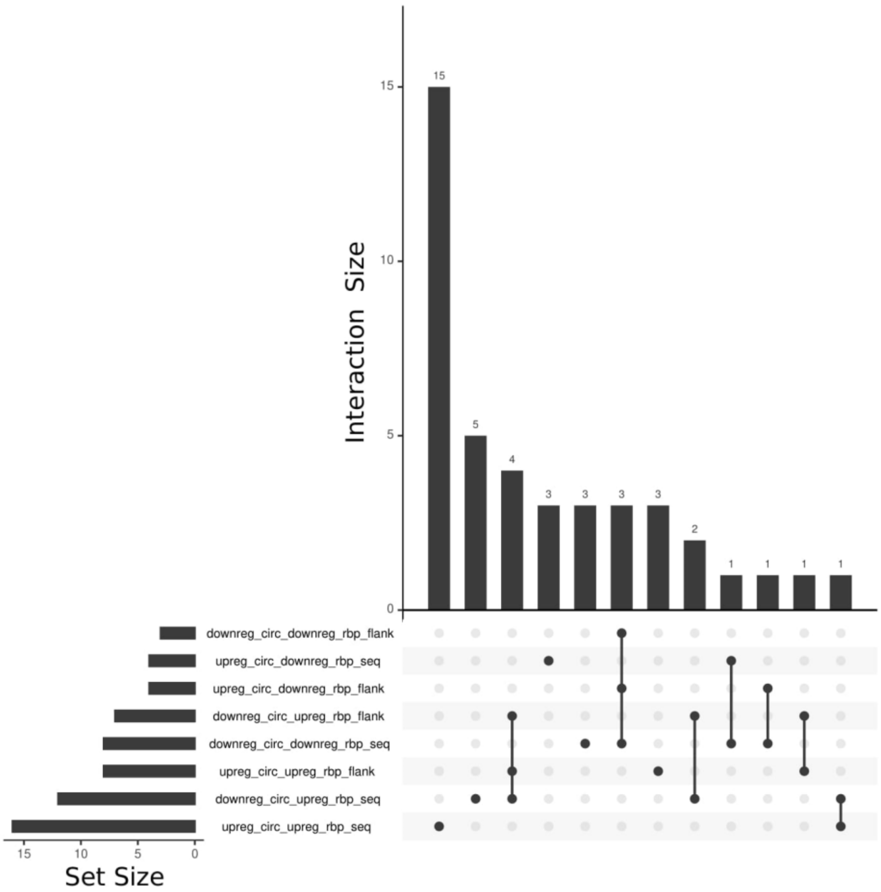

RBP											
**uC_uR_s***	**dC_uR_s***	**dC_uR_s*<br>uC_uR_f*<br>dC_uR_f***	**uC_dR_s***	**dC_dR_s***	**dC_dR_s*<br>uC_dR_f*<br>dC_dR_f***	**uC_uR_f***	**dC_uR_s*<br>dC_uR_f***	**dC_dR_s*<br>uC_dR_s***	**dC_dR_s*<br>uC_dR_f***	**uC_uR_f*<br>dC_uR_f***	**uC_uR_s*<br>dC_uR_s***
EIF4G2	FUBP3	RBMS2	EIF4B	CPEB1	CPEB3	HNRNPR	RBM41	RBM24	ELAVL3	PABPC1	MBNL1
FUS	HNRNPD	SF3B4	HNRNPH2	ELAVL4	ELAVL2	IGF2BP2	RBM47				
HNRNPA2B1	PTBP1	TIA1	PCBP4	PUF60	KHDRBS2	IGF2BP3					
ILF2	RC3H1	ZFP36									
PCBP2	ZNF326										
PPRC1											
RBM25											
RBM4											
RBM6											
RBMX											
SRSF1											
SRSF11											
SRSF2											
SRSF4											
ZC3H10											

***dC_dR_s** – downregulated circRNA, downregulated RBP with motifs enriched in circRNA sequences
***dC_uR_s** – downregulated circRNA, upegulated RBP with motifs enriched in circRNA sequences
***uC_dR_s** – upregulated circRNA, downregulated RBP with motifs enriched in circRNA sequences
***uC_uR_s** – upregulated circRNA, upregulated RBP with motifs enriched in circRNA sequences
***dC_dR_f** – downregulated circRNA, downregulated RBP with motifs enriched in circRNA flanking introns
***dC_uR_f** – downregulated circRNA, upregulated RBP with motifs enriched in circRNA flanking introns
***uC_dR_f** – upregulated circRNA, downregulated RBP with motifs enriched in circRNA flanking introns
***uC_uR_f** – upregulated circRNA, upregulated RBP with motifs enriched in circRNA flanking introns

Supplementary Figure 14. Upset plot for RNA-binding proteins (RBPs) with binding motifs in flanking introns vs RBPs with binding motifs in circular RNAs (circRNAs).

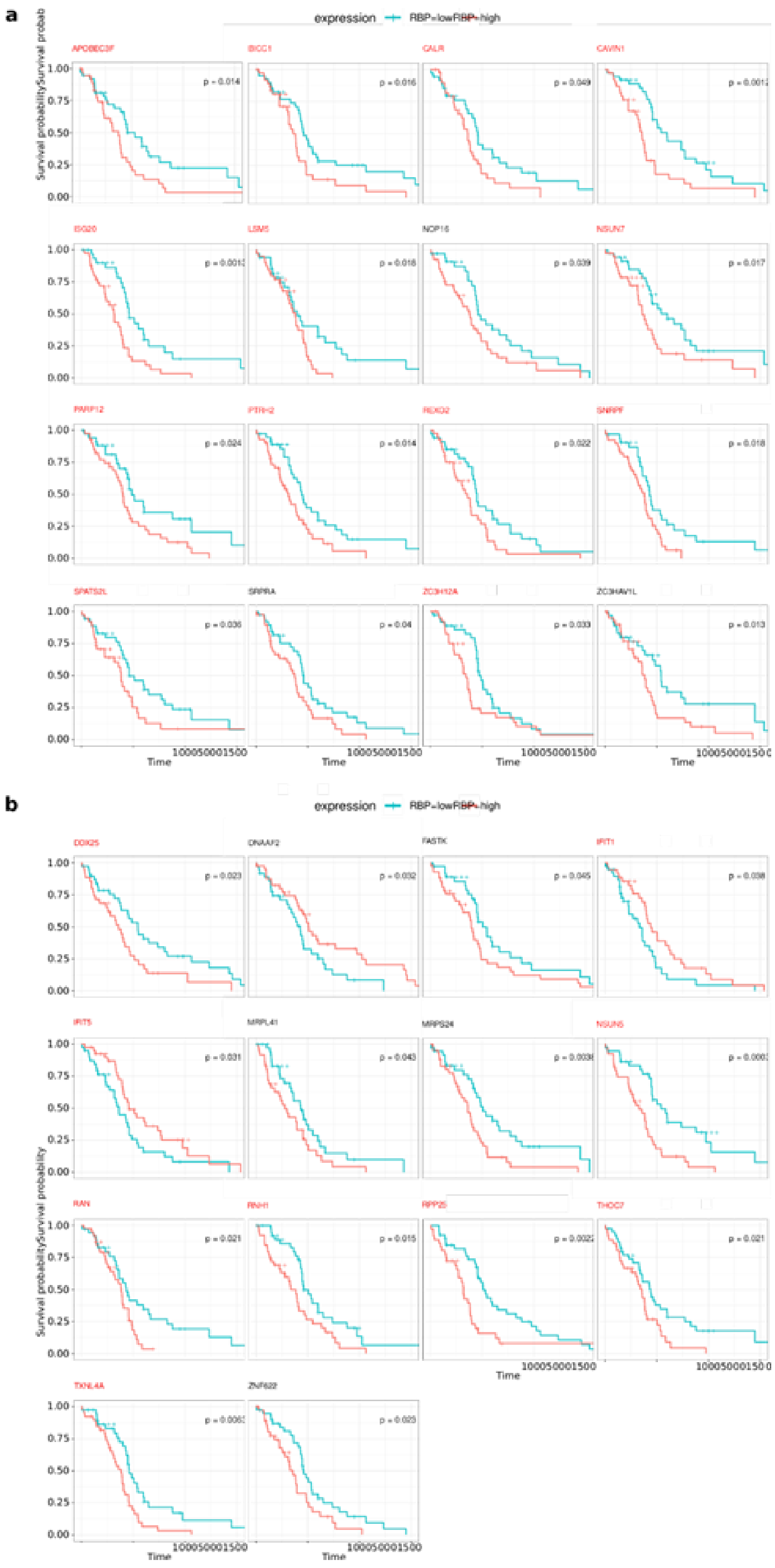

Supplementary Figure 15. Survival analysis for differentially expressed RNA-binding proteins (RBPs) based on TCGA glioblastoma (GBM) dataset. A. Kaplan-Meier curves for RBPs upregulated in analyzed 23 GBM samples and significantly correlated with overall survival rate with TCGA GBM dataset. **B.** The same as A, for downregulated RBPs. RBPs highlighted red in both panels were previously shown to be brain tumor markers, correlated with disease outcome or response to treatment.
